# When dates disagree: radiocarbon and sighting-based models reveal range-wide variability in great auk (*Pinguinus impennis*) extinction estimates

**DOI:** 10.1098/rsbl.2026.0223

**Published:** 2026-11-04

**Authors:** Lucia S. Snyderman, Kevin R. Burgio, Arthur W. Anderson, Olivia L. Olson, Samuel T. Turvey, Alexis M. Mychajliw

**Affiliations:** ^1^ School of Archaeology, Geography and Environmental Science, University of Reading, Reading, UK; ^2^ Institute of Zoology, Zoological Society of London, London, UK; ^3^ Department of Biology, Middlebury College, Middlebury, VT, USA; ^4^ Program in Environmental Studies, Middlebury College, Middlebury, VT, USA; ^5^ Northeast Climate Adaptation Science Center, University of Massachusetts Amherst, Amherst, MA, USA; ^6^ School of Criminology, Society, and Politics, University of New England, Biddeford, ME, USA; ^7^ Lake Champlain Maritime Museum, Vergennes, VT, USA

**Keywords:** extinction timing, radiocarbon, conservation palaeobiology, historical extinction, great auk (*Pinguinus impennis*)

## Abstract

Methods for inferring extinction are well established for events that either occurred on millennial time scales (e.g. the late Quaternary) or are unfolding in the present. However, these methods employ distinct data types inherent to palaeontological or neontological settings, yielding a potentially disjointed view of the extinction process. We leveraged the historical extinction of the great auk (*Pinguinus impennis*)—a large, flightless seabird native to the Atlantic—as a test case to assess the effects of using varied data types (286 quality-scored occurrences) and models (Gaussian-resampled inverse-weighted McInerny, Solow and Beet) in inferring extinction across centennial time scales. While the last confirmed great auk was killed in 1844 CE in Iceland, the dynamics of species-wide range collapse in response to differing harvest practices have not been explored. Our sighting-based estimates suggest the species may have persisted a decade longer (1860 CE) in the Northwest Atlantic, highlighting the need for future research on this understudied portion of their range. Radiocarbon-based models estimate extinction later (post-2000 CE) across the species’ range, suggesting that inherent uncertainties in radiocarbon dates and their calibration—particularly for marine samples—may constrain their applicability for detailing extinction processes on conservation-relevant time scales.

## 1. Introduction

The potentially lingering presence of a lost species can be both captivating and haunting [1,2]. The International Union for the Conservation of Nature (IUCN) defines extinction as when ‘there is no reasonable doubt that the last individual has died’ following comprehensive surveys across the species’ historical range [3]. Accurately determining when a species no longer exists is surprisingly difficult, yet doing so is critical for allocating conservation resources efficiently [4]. Incorrectly classifying a species as extinct when it is still extant (e.g. the ‘Romeo error’ [5] or the Signor–Lipps effect [6]) can erode public confidence in conservation science when that species appears to ‘rise from the dead’ (i.e. become a Lazarus taxon [7,8]). Despite the use of increasingly sophisticated detection models and monitoring technologies, such errors have occurred for more than 100 species of vertebrates [9–11].

The fossil record is an archive comprised of countless iterations of the extinction process, but direct comparison of past extinctions with ongoing losses remains complicated by the different data types and time scales of mass extinctions [12]. Even on the millennial time scales of the Late Pleistocene, our ability to translate chronological information from palaeontological and zooarchaeological deposits to present-day population processes is hindered by time-averaging, taphonomic biases [13] and the inherent uncertainty associated with radiocarbon dates. For example, application of standard radiocarbon-based models to extinct species in the Late Holocene (e.g. Gaussian-resampled inverse-weighted McInerny method (GRIWM)) can produce unrealistic projections of persistence that extend into the present or even the future [14–18]. The emerging subfield of conservation palaeobiology seeks to harness lessons from the past to disentangle the drivers of extant species decline and inform conservation decision-making but has yet to fully address these translational constraints [19–21].

Centennial time scales represent an opportunity to evaluate how methods for detecting extinction timing may converge or diverge in their results and utility, and to provide a bridge to encompass the full history of biodiversity change from the late Quaternary to the present [22,23]. Species that have become extinct during the recent historical period are both temporally closer and more familiar to us, but nonetheless have uncertainties surrounding their extinction given the time scales inherent to extinction as a process and the shifting baselines of human memory [24,25]. Advances in Bayesian sighting-based models have enabled the use of dated historical occurrence data to model the loss of species such as the Carolina parakeet (*Conuropsis carolinensis*), thylacine (*Thylacinus cynocephalus*) and ivory-billed woodpecker (*Campephilus principalis*) [26–28]. Here, we compare the information-content of different data types available for understanding biodiversity loss in the historical era using the historical extinction of the great auk (*Pinguinus impennis*).

The great auk was a large (approx. 3.5 kg) flightless seabird that occupied islands and coastlines across the North Atlantic [29]. Although it resembled a penguin (Spheniscidae), molecular data support a sister relationship to the extant razorbill (*Alca torda*) within the Alcidae. The oldest *Pinguinus* fossils date from the early Pliocene [30], and the taxon was widespread in the late Quaternary before its historical range collapse, with subfossil remains known from Norway to the Mediterranean in the Northeast Atlantic region, and from Greenland to Florida in the Northwest Atlantic [31–34]. Other flightless alcids (the Miocene–Pleistocene mancalline auks) are extinct [30], and nearly a third of extant alcids are now threatened with extinction [35].

While the great auk was first pursued for its meat, by the 1700s CE, the species was rapidly overharvested for the European feather trade [36]. The last confirmed capture occurred on Eldey Island, Iceland, in 1844 CE [37]. However, coeval accounts also describe an individual being mistaken for a storm-causing witch off St Kilda, Scotland, around 1844 CE [38] and the IUCN accepts a later 1852 CE report in Newfoundland as probably genuine [35]. Sighting records reflect increased contemporary public interest in the species, with unconfirmed sightings persisting until 1870 CE in Iceland [39,40] and Labrador [41], possibly suggesting that these regions could serve as refugia for remaining individuals. The spatiotemporal pattern and quality of the nineteenth-century great auk reports across the Northeast Atlantic have not been systematically assessed, and no studies have evaluated comparative extinction timing in the Northwest Atlantic. Here, we present the first comprehensive dataset of radiocarbon and historical sighting dates across the species’ Holocene range. In particular, we include data from the Gulf of Maine (Northwest Atlantic), a region overlooked in previous studies [42]. We use these data to assess the complementarity of palaeontological and neontological approaches [14,28] to estimate extinction timing across the species’ range and for discrete population units (figure 1).

**Figure 1.**
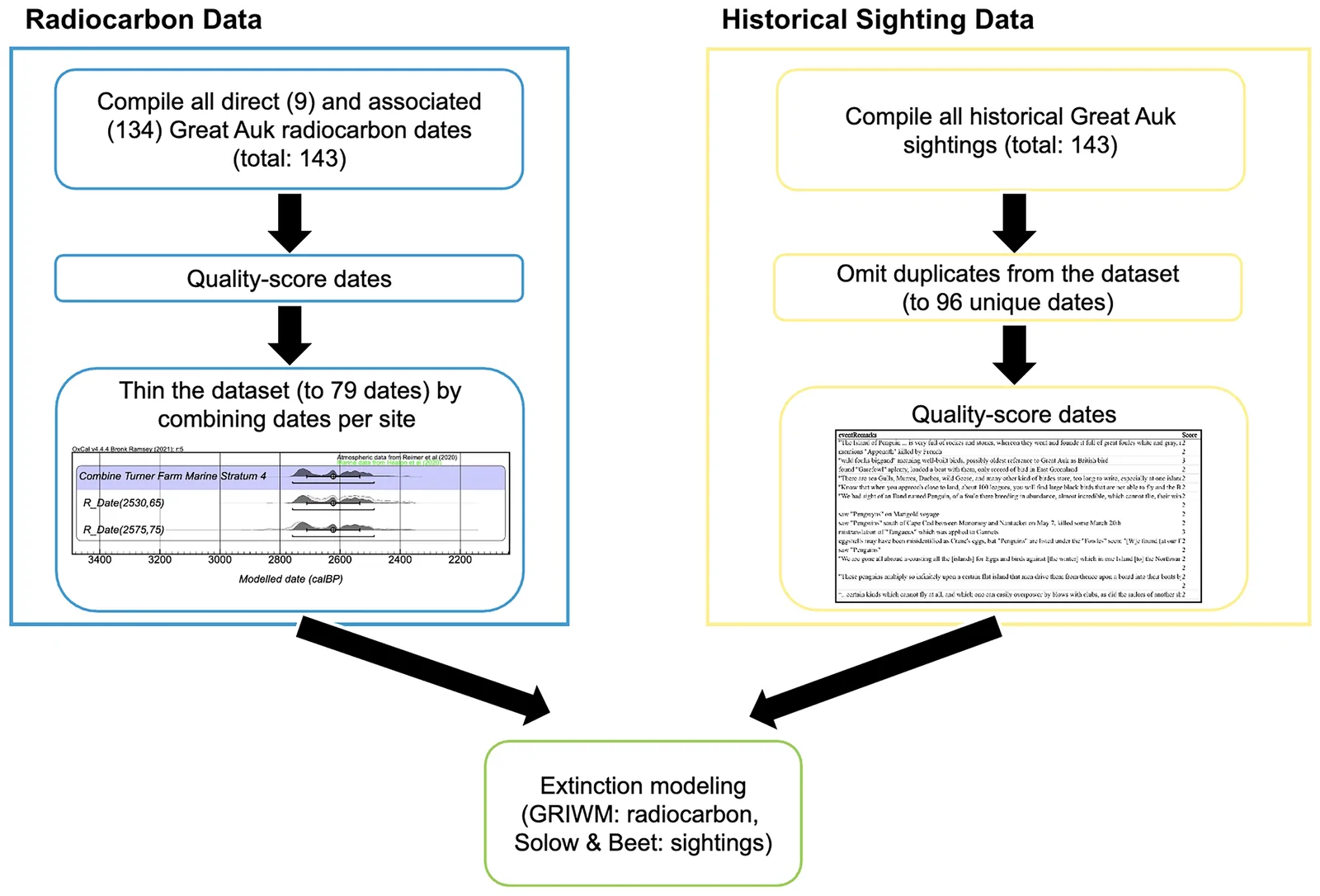
Schematic of the methodological workflow employed in this study.

## 2. Material and methods

### (a) Radiocarbon database curation

We compiled 143 radiocarbon dates from previous studies. Sources included the Canadian Archaeological Radiocarbon Database (CARD) [43], the Paleobiology Database (PBDB) [44] and published literature and archaeological reports. We developed a standardized quality ranking system as modified from [45,46] and assigned quality scores to all dates based on associative strength and stratigraphic control, reliability of dated material and dating method, ranging from 3 (lowest score) to 13 (highest score) (table 1).

**Table 1.** Numerical ranking scale for assessing radiocarbon dates employed in this study (as modified from [45,46]).

(a) material dated	score
bone collagen	6
charcoal or wood	5
soil	3
marine shell	2
trace organics, whole bone or unknown	1
**(b) strength of association**	
date on specimen itself	5
date from specimen stratum or feature	3
date from stratum near specimen stratum	2
date from associated cultural period or unknown stratum	1
**(c) radiocarbon dating method**	
accelerator mass spectrometry	2
standard (beta) or unknown	1

The majority of radiocarbon dates available were associated with great auk remains; only nine dates are available on great auk bone (supplementary material, figure S1). Further, at thoroughly excavated sites, the number of associated dates was not equivalent to the number of great auk specimens per stratum and instead reflected other research priorities of the original excavators. Thus, we assessed the effect of this variability in abundance of dated material per great auk-bearing site by thinning our dataset to avoid overrepresenting observations of great auks in the fossil record in our extinction models [47] and present both thinned and unthinned results. For these sites, in OxCal v.4.4 [48], we modelled each stratum's boundary start and end dates, used the ‘Combine’ function if there were fewer than three dates per stratum and omitted loosely associated dates (e.g. material only dated to the same cultural period) [49] (see supplementary material for detailed discussion and code).

We calibrated terrestrial dates using the IntCal20 calibration curve [50]. To calibrate marine material, we applied the Marine20 calibration curve and selected appropriate local reservoir corrections [51] based on site coordinates (as found in the literature or using GEOLocate v.3.22 [52]), and estimated weighted mean ΔR and uncertainty using the 10 nearest coordinates. This resulted in a final radiocarbon dataset of 79 thinned dates from 28 sites across the species’ Holocene range (Northwest Atlantic: 74 total, 45 thinned; Maine: 59 total, 34 thinned; Northeast Atlantic: 69 total, 34 thinned) (figure 2). All direct great auk radiocarbon dates (nine) were from the Northeast Atlantic.

**Figure 2.**
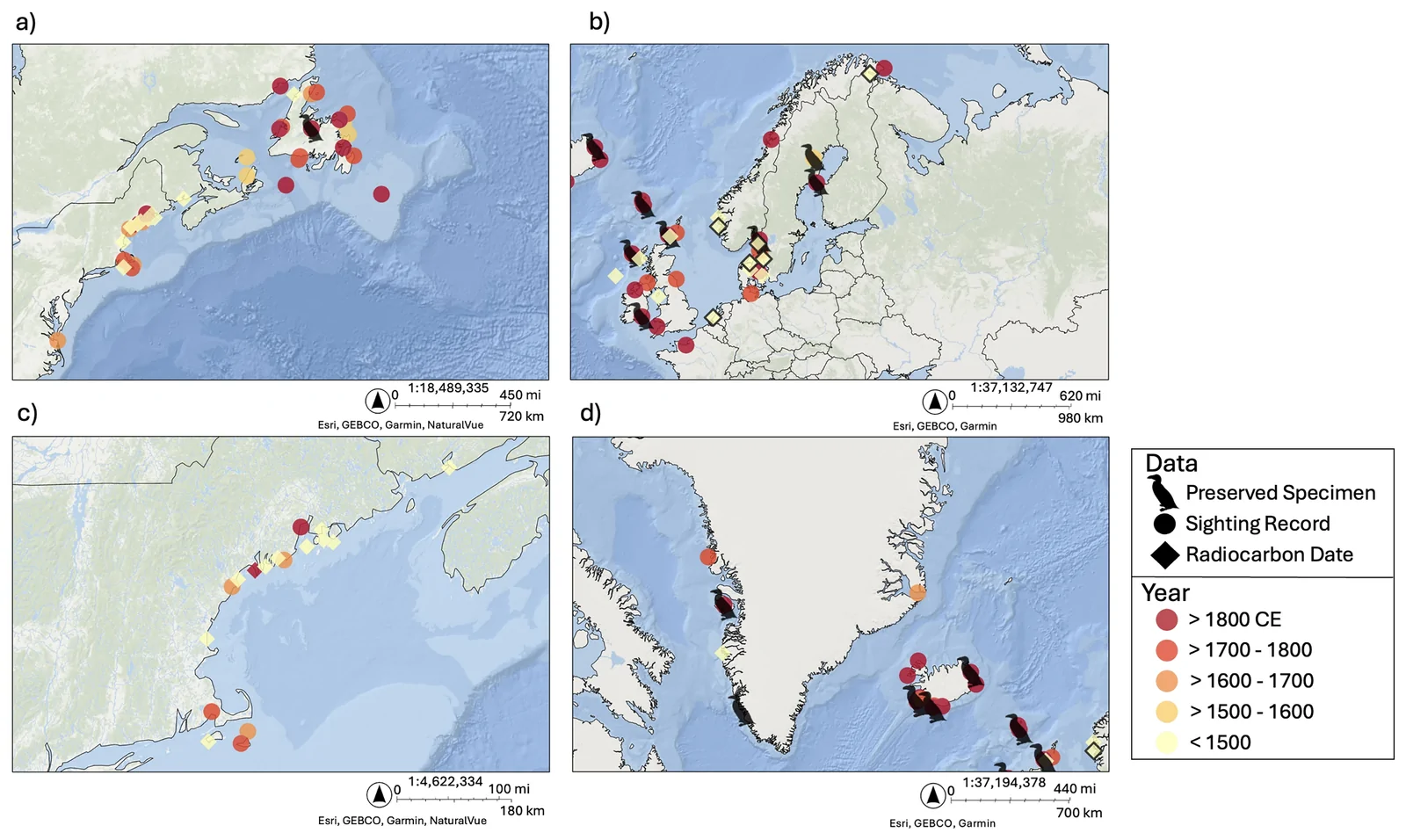
Great auk spatiotemporal occurrence for the following regions: (a) Northwest Atlantic, (b) Northeast Atlantic, (c) Gulf of Maine and (d) Greenland and Iceland. One radiocarbon date from Morocco (6135 ± 209 cal ybp; UQ-1601 [53]) is not displayed in the (b) panel owing to geographic scope. Radiocarbon dates include associated and direct dates, with the latter displayed with black outlines. Maps made in ArcGIS Online. Great auk silhouettes from PhyloPic [54].

### (b) Radiocarbon-based extinction modelling

We employed the GRIWM model [18] to estimate extinction timing. This approach accounts for variation in temporal fossil distribution and uncertainty in radiocarbon dating using a down-weighting interval procedure. The newer version of the model, the calibration-resampled inverse-weighted McInerny method (CRIWM) [15], cannot calibrate marine dates from different ocean basins in the same model run. We implemented CRIWM for comparative purposes, which required us to remove all marine dates from our dataset—including all directly dated great auk material—and introduces a bias in occurrence sampling (supplementary material, table S2). We recognize this is a limitation of currently available and commonly used radiocarbon-based extinction estimation models, which has been noted in previous research on wide-ranging marine taxa [55].

We estimated extinction timing for the great auk's entire range and for specific populations: (i) Northwest Atlantic population (Canada, Greenland, Gulf of Maine, United States); (ii) Gulf of Maine in isolation; and (iii) Northeast Atlantic population (Denmark and Faroe Islands, France, Germany, Iceland, Morocco, Netherlands, Northern Ireland, Norway, Republic of Ireland, Sweden, United Kingdom). We also separately analysed all high-quality dates (score greater than or equal to 9), all low-quality dates (score less than 9) and the high-quality dates for each population to assess biases between high-quality and low-quality dates. We repeated all analyses to assess the effects of our geographic, thinning and quality subsets (see supplementary material, table S1). All analyses were conducted in R version 4.3.3 [56].

### (c) Historical sighting database curation

We assembled a historical sighting database by searching literature including historical whaling archives, testimonies and diaries in print and online in several languages, as well as by collating museum records from the Global Biodiversity Information Facility [57]. We searched for great auk sightings under 89 alternative names and spelling variations (e.g. the Icelandic name ‘geirfugl’ or ‘spear-bird’) [40] (table 2). We used GEOLocate [52] to consistently determine geographic coordinates and assigned quality scores (1–3) to all records based on their credibility: (1) specimen collected at a known date, (2) confirmed sighting without an associated physical specimen, and (3) unconfirmed sighting [23] (table 3).

**Table 2.** Names used for the great auk across its historical North Atlantic range.

name(s)	locality
abâktoo'e-âk	Maine
*Alca impennis*	entire range
*Alca borealis*	Sweden
alk(a)	Scottish isles
an erbhoil/An Gearbhul/An Gearra-bhul	British Isles
anglema(a)ge/Angledemager/Anglemager	Denmark
*Anser Magellanicus, seu Pinguini*	Denmark
ap(p)onar/t	Newfoundland
aponas	Newfoundland
aporath	Newfoundland
apponath/Apponatz	Newfoundland
auk	Scottish isles
avis-gare	Scotland
avis garfahl	Sweden
barricardières	France
binocle	North America
bonnivochil/bishop-fowl	Scotland
brillefugl	Denmark
brillenalk	Denmark
bunnabhuächaille	Scotland
*Chenalopex impennis*	British Isles
der riesenalk	Germany
duke	Spain/France/Newfoundland
esarokitsok/isarukitsok/iserokitsok/isorakitsoq	Greenland
fiaert	Norway
f(l)ügloser alk	Denmark
gaarfugl(ur)	Faroe Islands
gairfowl	St. Kilda, Scotland
garefowl(e)	Scotland/Republic of Ireland
garfågel	Sweden
garefogel	Germany/Faroe Islands/Sweden
garfahl	Sweden
garfowl	Scandinavia/Scotland
garf(u/o)gel	Faroe Islands
gårfuglur	Faroe Islands
gayrfowl	St Kilda, Scotland
gearrbhul	British Isles
geir	Iceland
geirfugl	Scandinavia
geirvogel	Germany
geyerfogel	Canada
geyrfugl	Iceland
goi(r)fug(e)l	Faroe Islands/Scotland
gorfou	France
gorfuglir	Faroe Islands
le grand pingo(u)in/grand pingouin du nord	France
great auk	entire range
grosser alk	Germany
grosser papageitaucher	Germany
g(e)yrfugl/gyrvgeyrfugl	Iceland
havtirdill	Iceland
king and queen of the auks	Papa Westra, Orkney, Scotland
kurzflügeliger alk/kurzflügeliger	Germany
large auk	Newfoundland
lengurn	Canada
*Matœoptera impennis*	Sweden
*Mergus americanus*	North America
moyack	Massachusetts/Labrador
noordscher penguyn	the Netherlands
nordischer fettgans/nordischer pinguin	Germany
nordske penguin	Sweden
northern auk	British Isles
northern penguin	unknown
ogaen	unknown
peegvie	Norway
penguin	entire range
penguyn	the Netherlands
pengwin	Cape Cod, Massachusetts
pengwyn	Cape Breton and Funk Island, Canada
pennego(u)in	Newfoundland, Canada
penngwyne	Newfoundland, Canada
pingouin (brachiptère)	France
pinguin-alk	Germany
*Pinguinus impennis*	Entire range
pingwin	Faroe Islands
pinwing	Newfoundland
*Plautus impennis*	Sweden
reuzenalk	the Netherlands
schwimmalk	Germany
sea-woggin/waggin	Newfoundland
storömmer	Norway
stummelalk	Germany
tangueu/tangueux	Canada
vulture	Iceland
w(h)obble	New England
wogæn/wogen/woggin/woging/woglin	North America
wuttoowaganash	Squibnocket, Massachusetts
yawgin	North America

**Table 3.** Example sighting quotes with assigned reliability scores (as modified from [26]).

specimen-based sighting (1)	confirmed sighting (2)	unconfirmed sighting (3)
‘It walked like a man … but moved its feet quickly … Its wings lay close to the sides—not hanging out. I took him by the neck and he flapped his wings. He made no cry. I strangled him’ (Alfred Newton's 1861 record of an 1844 report from a local Icelandic fisherman, Eldey Island, Iceland) [39]	‘Some of these birds are as large as geese, being black and white with a beak like a crow's. They are always in the water, not being able to fly in the air, inasmuch as they have only small wings about the size of one's hand … And these birds are so fat that it is marvelous …’ (Jacques Cartier, 1534, Funk Island, Canada) [58]	‘Johannes Propert shot a bird which he had never seen before, but which, from description, could be no other than the Great Auk … Johannes bears the reputation of telling wonderful tales now and then’ (Friend of Johannes Propert, 1859, Disko Island, Greenland) [59]

### (d) Sighting-based extinction modelling

We analysed historical sighting data using a Bayesian extinction timing model [60] which accounts for sighting quality, with specimen-based and confirmed sightings having a greater impact on the extinction date estimate (τ*_E_*) than unconfirmed sightings. Here, *p*(*t*|τ*_E_*) is an expression for the likelihood that extinction occurred at a given date, where ‘E’ is the event that the species is extinct by a given year, and ‘t’ is the sighting dataset. We implemented Solow and Beet's ‘Model 2’ in R [26,28,61] using a uniform prior and 1870 CE as the end of our observation period (see supplementary material).

To evaluate whether specimen-based and unconfirmed sightings produce different temporal extinction patterns, we conducted combined analyses of specimen-based and confirmed sightings (scores 1+2) and confirmed and unconfirmed sightings (scores 2+3). If there were more than one sighting per year, we omitted duplicate sightings based on reliability, keeping the highest-scoring date. We also re-ran the model to estimate extinction using the earliest, median and latest dates when reports provided a range of possible dates. We estimated extinction timing for the species’ entire range and for designated populations with sufficient sample sizes: the Northwest Atlantic and the Northeast Atlantic (see supplementary material).

## 3. Results

Radiocarbon-based extinction timing analysis (GRIWM) across both the entire range of geographic subsets typically produced an estimated median extinction date post-1950 CE with confidence intervals that included the nineteenth century and extended into the future (supplementary material, table S1), consistent with GRIWM results of other Late Holocene extinctions [14–18,62]. For example, unthinned and thinned range-wide estimates are 2037 CE (95% CI: 1826–2234 CE) and 2137 CE (95% CI: 1853–3184 CE), respectively. For these same bins but only including terrestrial dates, CRIWM yielded only extinction estimates that pre-date the last known occurrence of the species (supplementary material, table S2). When the radiocarbon dataset was subset by data quality, unthinned high-quality dates (scores greater than or equal to 9) across the entire range yielded an early extinction estimate of 1416 CE (95% CI: 1250–1641 CE) (supplementary material, figure S2). Using unthinned high-quality dates only for specific populations provided the following extinction estimates: the Northwest Atlantic estimate is 1420 CE (95% CI: 1240–1657 CE), the Maine estimate is 1419 CE (95% CI: 1239–1667 CE) and the Northeast Atlantic estimate is 2448 CE (95% CI: 2162–2717 CE) (figure 3; see supplementary material). Differences in extinction estimates on the scale of several hundred years might be inconspicuous for extinctions that occurred on the scale of thousands of years in the past (i.e. in the Pleistocene [64–66]), but appear more pronounced when placed into historical contexts [14,16,67].

**Figure 3.**
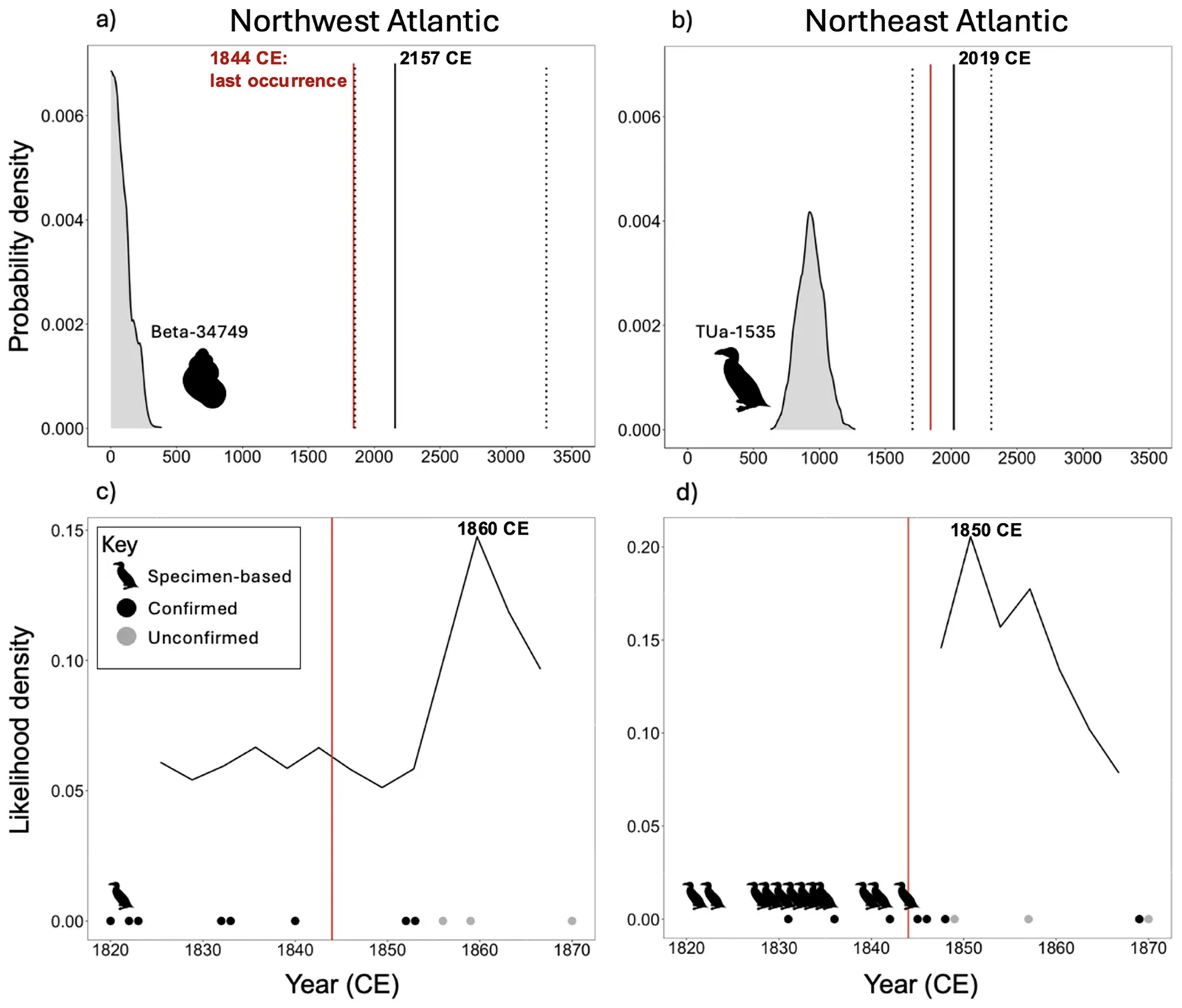
Radiocarbon-based (GRIWM) and sighting-based thinned extinction estimates for the Northwest Atlantic and the Northeast Atlantic great auk populations (supplementary material, table S1). The most recent calibrated radiocarbon dates in OxCal with the Marine20 calibration curve from sites in Maine ‘The Basin’ (124 ± 86 cal ybp; beta-34749 [63]) and Norway ‘Kobbehelleren’ (1105 ± 100 cal ybp; TUa-1535 [34]) are plotted in the (a) and (b) panels. Panels (c) and (d) show the likelihood density on the *y*-axis and displays the posterior probability of a given extinction date, τ*_E_,* for the great auk (based on Solow & Beet's model 2 [26,28]): the Northwest τ*_E_* is 1860 CE (95% CI: 1825–1867 CE, *p*(*t*|τ*_E_*) = 2.21 × 10^56^) and the Northeast τ*_E_* is 1851 CE (95% CI: 1848–1867 CE, *p*(*t*|τ*_E_*) = 2.00 × 10^57^). Red lines represent the last confirmed sighting in 1844 CE. Black solid lines represent extinction estimates generated from the thinned population datasets, and black dotted lines represent error ranges.

Of the 143 historical sightings across the great auk's range, 96 are unique and date from 1527 to 1870 CE (figure 2). The Northeast Atlantic had more total reported sightings (*n* = 75) and specimen-based sightings (scored 1; *n* = 17) than the Northwest Atlantic (total, *n* = 55; specimen-based, *n* = 3), with only two historical sightings from Maine (supplementary material, figure S3). Our historical sighting dataset contains seven low-quality unconfirmed sightings pre-1800 CE and 13 post-1800 CE, suggesting that early records of the species from the period when it is known to have been extant are not necessarily of higher quality than later records from the period when the species was declining and hard to detect, or that may represent potential misidentifications post-dating its likely extinction [60,62]. Specimen-based and confirmed sightings (scores 1+2) for the entire range generated an extinction date estimate of 1848 CE (95% CI: *p*(*t*|τ*_E_*) = 8.15 × 10^111^), and the combined analysis of confirmed and unconfirmed sightings for the entire range (scores 2+3) produced an extinction date estimate of 1860 CE (95% CI: 1846–1867 CE, *p*(*t*|τ*_E_*) = 6.97 × 10^111^). Range-wide analysis of all sighting dates (95% CI: 1846–1867 CE, *p*(*t*|τ*_E_*) = 2.72 × 10^128^) and Northwest Atlantic population-level analysis (95% CI: 1825–1867 CE, *p*(*t*|τ*_E_*) = 2.21 × 10^56^) recovered the same estimated τ*_E_* of 1860 CE, whereas the Northeast Atlantic population was estimated to have gone extinct a decade earlier in 1851 CE (95% CI: 1848–1867 CE, *p*(*t*|τ*_E_*) = 2.00 × 10^57^) (figure 3). However, error ranges for all these estimates overlap substantially.

## 4. Discussion

Nearly a third of extant seabird species are classified as globally threatened and many have gone extinct historically [35]. Perspectives derived from palaeontological data can contextualize contemporary trends of range change and collapse to identify possible intervention points, as well as illuminate potentially shifted baselines in the structure and function of ecosystems [68,69]. Here, we describe estimates of extinction and range contraction for great auks and highlight important data gaps that need to be bridged by future conservation palaeobiology approaches in extinction research.

Extinction timing is rarely uniform across a species’ range, with differences emerging even between subpopulations [70]. Despite the popular story that the last great auk was killed in Iceland in 1844 CE, our modelled sighting data suggest that populations in the Northwest Atlantic, including the Gulf of Maine, may have persisted longer than the Northeast Atlantic population (figure 3). Our preliminary findings lay a foundation for future hypothesis testing regarding this population's persistence and whether it could be due to differences in harvest practices. In the Northwest Atlantic, Indigenous communities including the Inuit in Greenland [71] and the Beothuk in Newfoundland [72] had relationships with the great auk for millennia. By the eighteenth century, however, Europeans had begun large-scale harvesting of great auks to supply the rapidly growing demands of the feather trade [36]. Populations may have persisted longer in habitat refugia in North America, particularly the Gulf of Maine, owing to a delay in the arrival of European exploitation pressures [73].

Accurately disentangling true variation in geographic patterns of range collapse from sampling biases in historical and Quaternary records can be challenging [74–76]. Our historical sighting dataset shows a spatial bias towards more ‘accessible’ regions, suggesting that great auks may have persisted in remote regions without being recorded. For example, Eldey Island, the site of the last confirmed great auk capture, was relatively accessible to hunters from the adjacent Reykjanes Peninsula; this region is close to Iceland's capital and was also easily visited by nineteenth-century scientists [37]. A comparable spatiotemporal bias is also shown by the fact that nearly all sightings are from breeding sites during the May–June breeding season [77]. These spatial biases highlight the unexplored potential for relictual persistence of the species in the Northwest Atlantic.

We leveraged the opportunity afforded by the Holocene to assess the congruence of extinction estimates provided by neontological and palaeontological approaches. Radiocarbon dates are typically used in inferring the timing of Late Pleistocene extinctions and sighting observations are used for historical extinctions, with each data source typically used in isolation. However, both data types are potentially available in parallel to investigate extinctions occurring in the Late Holocene [78], and can thus be used to cross-check each other. If one goal of the emerging subfield of conservation palaeobiology is to use Late Pleistocene extinctions to provide insights into modern conservation dilemmas, the time-averaging and ranges of uncertainty generated by palaeontological methods must be made translatable to conservation issues in the present [79]. Our study reveals that while radiocarbon dates offer valuable insights into species’ spatiotemporal occurrences and can be used to reconstruct the timing of late Quaternary extinctions [80], they seem to constitute an imprecise tool for determining extinction timings at the temporal resolution of the historical era due to their associated centuries-wide calibration distributions; this issue is exacerbated by marine reservoir effects [51]. The majority of available great auk radiocarbon dates are more than a thousand years old, reflecting a sampling bias in archaeology; historical sites are typically aged by correlations with cultural context, documents and objects such as ceramics [81]. High-quality radiocarbon dates for great auks were available only for older material, yielding extinction estimates that pre-date confirmed historical observations and most historical extinction estimates. Conversely, low-quality radiocarbon dates were generally produced on more recent material, with associated extinction estimates extending into the 2000s CE. GRIWM has been known to produce anomalously recent extinction dates and confidence limits that extend into the future for several taxa (i.e. are negative model outputs), notably for those that are either very poorly sampled with a small number of radiocarbon dates spaced widely in time and/or for species with Late Holocene extinctions close to the present with radiocarbon dates that have large uncertainty ranges [14–17]. Such wide uncertainties are also expected when considering radiocarbon dates from marine samples, given issues associated with offsets and reservoir corrections [51,55]. While such uncertainties are reasonable on the scale of events across millennia or centuries, such as for late Quaternary megafauna, they can complicate understanding of historical events where decadal resolution is important. The specific extinction outputs of our radiocarbon-based models represent a first step in estimating great auk extinction timing, which should be refined in the future as more radiocarbon dates are generated for the Northwest population and more sophisticated models become available that can better incorporate marine reservoirs.

Radiocarbon dates can have centuries-wide distributions when calibrated, yet present-day extinction evaluations are based on, at most, decades of surveys [4]. This broad temporal uncertainty range can hinder our ability to correlate extinctions with specific historical events or policy changes [78]. To make the most of extinction lessons from the past, we recommend that complementarity, information-content and ecological relevance be investigated across multiple different environmental archives to establish new baselines across the Holocene. We also call for further development in radiocarbon-based models of extinction that can incorporate variation across the full geographic range of marine taxa and overall greater collaboration in methods development between palaeontologists and conservation biologists.

## Supplementary Material



## Data Availability

The datasets supporting this article have been uploaded as part of the supplementary material and can also be found on GitHub [82]. Supplementary material is available online [83].
