# Supplementary material for: When dates disagree: radiocarbon and sighting-based models reveal range-wide variability in great auk (Pinguinus impennis) extinction estimates
Source: Biol Lett. 2026 Nov 4;22(11):20260223. doi: 10.1098/rsbl.2026.0223 (PMC13730643; doi:10.1098/rsbl.2026.0223)
Supplement: Supplementary file 1 [file rsbl.2026.0223.zip › rsbl.2026.0223/rsbl20260223_si_001.docx]

**Electronic Supplementary Materials**

Snyderman, L., Burgio, K., Anderson, A., Olson, O., Turvey, S., Mychajliw A. “**When dates disagree: radiocarbon and sighting-based models reveal range-wide variability in Great Auk (*Pinguinus impennis*) extinction estimates**”, submitted to Biology Letters, 2026.

Please also see GitHub for additional information and easily downloadable files that contain raw data (e.g., .csv) for radiocarbon dates and historical sightings: <https://github.com/luciasnyderman528/Great-auk-radiocarbon-historical-sighting-dataset>

Contents

This Electronic Supplementary Material document contains the following text:

1. Radiocarbon-based methods & outputs
   a. Database curation and calibration
   b. Thinning in OxCal
      i. Supplemental Figure 1
   c. Considerations for specific zooarchaeological sites with multiple dates
   d. Radiocarbon-based extinction modeling with GRIWM
      i. Supplemental Table 1
      ii. Supplemental Figure 2
   e. Radiocarbon-based extinction modeling with CRIWM
      i. Supplemental Table 2
2. Sighting-based methods & outputs
   a. Historical sighting database curation
      i. Supplemental Figure 3
   b. Solow & Beet extinction modeling
      i. Supplemental Figure 4
      ii. Supplemental Figure 5
3. References

Appendix:

1. OxCal code for use of Combine
2. Table of Great Auk names and their associated references

1. Radiocarbon-based methods & outputs

*(a) Database curation and calibration*

We compiled a comprehensive dataset of 143 Great Auk radiocarbon dates from previous published studies, including 134 associated dates (mainly generated on terrestrial material, particularly charcoal) and nine dates generated directly from Great Auk bones (see “Radiocarbon Dataset.xlsx”). An associated date is a radiocarbon date on material in the same stratum of a shell midden that also has Great Auk bones, and so they roughly correspond to the same age. Sources included the Canadian Archaeological Radiocarbon Database (CARD) [1], the Paleobiology Database (PBDB) [2], and original archaeological reports and the published literature.

We modified existing standardized quality ranking systems that account for differences in stratigraphic control and sampled material by modifying existing systems used in Late Quaternary studies [3;4]. We assigned quality scores to all dates based on associative strength, reliability of dated material, and dating method (accelerator mass spectrometry (AMS) or radiometric), ranging from three (lowest score) to 13 (highest score) (Table 1). For example, an AMS date on great auk bone collagen scored 13, whereas a standard date on plant material from a weak cultural period association scored three. For the state of Maine population specifically, we compiled 61 associated (60) and direct (1) radiocarbon dates on material from across nine Maine archaeological shell deposits. These dates span the Archaic and Ceramic/Woodland Periods or the Holocene (from ~8500 to 300 BP or 6550 BC to AD 1650), and the majority are from charcoal samples, but also include shells, soil, and bone. We excluded dates from sites in which there was known stratigraphic mixing of material as reported by authors of reports. Please see Supplemental Data Files for original radiocarbon dates, scores per date, and calibration information associated with each date.

We calibrated terrestrial dates using the IntCal20 calibration curve [5] using OxCal v.4.4 [6] and produced estimates of the mean ± σ, median, and 95.4% range (2σ interval). We applied the Marine20 calibration curve and local reservoir corrections [7] to calibrate marine dates by compiling latitude and longitude coordinates from the literature or GEOLocate v.3.22 [8] and estimated Weighted Mean ΔR and Uncertainty using the ten nearest coordinates. For example, a [Δ](https://en.wiktionary.org/wiki/%CE%94)R of -72 and ±61 corresponded to Casco Bay, Maine. Please see Supplemental Figure 1 for the distribution of terrestrial versus marine dates.

*(b) Thinning in OxCal*

To assess how variability in abundance of dated material associated with Great Auk per site may affect extinction timing estimates, we thinned our dataset to avoid oversampling, since at thoroughly excavated sites the number of associated dates was not equivalent to the number of Great Auk specimens per stratum e.g., [9]. For instance, Great Auk occurs throughout three different strata at the Turner Farm site in Maine, and is associated with a wide span of radiocarbon dates on marine and terrestrial material. In OxCal v.4.4 [6], we modeled each stratum’s boundary start and end dates by calibrating terrestrial and marine dates separately. We used the “Combine” function to combine if there were fewer than three dates per stratum. The “Combine” function in OxCal was designed specifically to be used in cases where different samples (that can have different reservoirs) have been radiocarbon dated that are coeval/drawn from the same historical event. Dates are first calibrated independently, then the overlapping probability density functions are combined and tested using the model agreement index (A_comb_), where A_comb_ ≥ A_n_ and A_n_ = 1/√2n and n = number of samples (see OxCal manual for further details). Please see the Appendix for the OxCal code used to perform this.

For the eight sites with the largest numbers of dates, we omitted loosely associated dates (e.g., material only dated to same cultural period), combined dates with the “Combine” function, and estimated phase boundaries (see below). All Combined dates passed the model agreement test (A_comb_). This resulted in a final radiocarbon dataset of 79 thinned dates from 28 sites across the species’ Holocene range (Northwest Atlantic: 74 total, 45 thinned; Maine: 59 total, 34 thinned; Northeast Atlantic: 69 total, 34 thinned) (see “Radiocarbon Dataset.xlsx”).

Supplemental Figure 1. All calibrated radiocarbon dates (i.e., the unthinned entire dataset across the full geographic range), with shapes indicating associated (circles) versus direct (triangles) dates (note there are only nine direct dates on Great Auks – all inherently marine given that Great Auks are seabirds) and colors indicating terrestrial (green) and marine (blue) calibrations (see text for details regarding calibration).

*(c) Considerations for specific zooarchaeological sites with multiple dates*

Please see below for specific details on the radiocarbon dates for each of the eight archaeological sites noted above with multiple dates:

*Turner Farm, Maine* [10]: The Turner Farm site presented challenges as there are three different strata representing Great Auk occurrence with a widespread span of radiocarbon dates, both marine and terrestrial. In OxCal, we modeled the boundary start and end dates of each phase or stratum by inputting and calibrating the terrestrial and marine dates separately. If there were less than three dates in a phase, we used the Combine function to combine the dates. For instance, Stratum 4 includes six terrestrial dates which were calibrated and modeled whereas the two other marine dates were calibrated and combined.

*Køtedalen, Norway* [11]: Starting with Phase 2, all five radiocarbon dates are terrestrial and so we calibrated and modeled the phase start and end dates. Phases 12, 13, 14, and 15 similarly consisted of only terrestrial dates and so the same procedure was followed.

*Tofts Ness, Scotland* [12]: This site has three phases with a total of nine dates [14]. Because there are only two, three, or four dates in a phase, we combined the dates in each phase. All the dates are terrestrial so there is one combined date per phase.

*Goddard, Maine* [13]: Because Great Auk is not found throughout the occupations of this site and rather directly co-occurs with specific features, we kept the eight directly associated dates separate and not combined.

*Great Diamond Island, Maine* [14]: Great Auk is associated with certain areas and strata at this site, but not specific features. Three terrestrial dates represent three different features that are all within Area B, Stratum III, but one of the dates is much earlier than the others. The two dates closer in time to each other were combined and the other date was kept separate. In Area C, Great Auk is represented by two different strata, and, as one date is terrestrial and the other is marine, we kept them separate.

*Frisby Butler, Massachusetts* [15]: Three terrestrial dates belong to Stratum 2 and were combined in OxCal, and one date was left out because it was four hundred years older than the others. One marine date was also kept separate. For Stratum 3, the two terrestrial dates were combined.

*Sejerø Island, Denmark* [16]: No specific stratum information was given for this site. Instead, Great Auk is associated with material found in certain excavations. Because this is a loose association, we ranked these seven dates low and modeled the two excavations together as a single phase to determine early and late boundaries. The two marine dates were kept separate.

*Hornish Point, Scotland* [17]: There is a mixture of terrestrial and marine dates at the site with Great Auk occurring in three different blocks. We took the middle date of three from Block 19 and kept the other two dates from Blocks 12 and 13 unmodeled.

*(d) Radiocarbon-based extinction modeling with GRIWM*

We applied the Gaussian-Resampled Inverse-Weighted McInerny Method (GRIWM) model [18], which uses a down-weighting interval procedure and thus more heavily weights younger radiocarbon dates closer to the last appearance date in the fossil record. This method is applied to calibrated radiocarbon dates and generates an estimate of extinction timing with a 95% confidence interval/band. This approach has been used in numerous published papers to estimate extinction timing, particularly for Late Quaternary megafauna (e.g., a major 2022 study of extinction in Southern California [19]). As we implemented GRIWM exactly using the R code provided by Saltré et al. 2012 [18] in the supplement of their published paper, we refer interested readers to their paper for download of this original code. We refer readers to our GitHub and supplemental data file for the exact .csv input files used for our GRIWM analyses in R, as executed in R version 4.3.3 [20].

GRIWM is intended to be run on calibrated dates and thus presents its output using calendar years before present (cal ybp), where the present in radiocarbon dating is defined as 1950 AD/CE (prior to the effects of nuclear testing on the atmosphere). Thus, the output of GRIWM R script is such that positive values are taken to indicate years before present dates (where 1950 is the present). Conversely, a negative value output indicates that an event occurred *after* present (i.e., these events occurred after 1950 CE) and thus would suggest that a species persists past 1950 CE [18]. As noted directly in the code “positive values indicate before present dates, e.g., 50 = 50 years BP; negative values indicate after day dates, e.g., -50 = 50 years AD” [18].

We assessed how sensitive GRIWM was to different subsets of data inputs by running the model numerous times with different inputs, including combinations of thinned vs. unthinned data, subsets of quality scoring (e.g., high vs. low quality ≥9 or <9), and varied geographic bins. We estimated extinction timing for the Great Auk’s entire range and for specific populations: (1) Northwest Atlantic population (United States, Gulf of Maine, Canada, Greenland); (2) Gulf of Maine in isolation; (3) Northeast Atlantic population (Iceland, Denmark and Faroe Islands, France, Germany, Morocco, Netherlands, Northern Ireland, Norway, Republic of Ireland, Sweden, United Kingdom) (Supplemental Table 1, Supplemental Figure 1).

Supplemental Table 1. All outputs from the Gaussian-Resampled Inverse-Weighted McInerney (GRIWM) extinction estimator [24] using the R code and the more recent Rextinct package provided by Herrando-Pérez and Saltré (2024) [27]. Note in GRIWM code that “BP” refers to before present, where 1950 = zero or present in radiocarbon dating, and “AD” refers to after day, time after the present of 1950 (*not Anno Domini*).

| **Region** | **Run** | **Lower (output)** | **Lower (CE)** | **Median**  **(output)** | **Median (CE)** | **Upper (output)** | **Upper (CE)** | **Sample Size** | **Filename (.csv)** |
| --- | --- | --- | --- | --- | --- | --- | --- | --- | --- |
| **Entire Range** | Unthinned, all | 124 / 124 BP | 1826 CE | -87 /  87 AD | **2037 CE** | -284 / 284 AD | 2234 CE | 143 | GRIWMEntireRange |
|  | Thinned,  all | 97 /  97 BP | 1853 CE | -187 / -187 AD | **2137 CE** | -1234 / 1234 AD | 3184 CE | 79 | GRIWMthinned |
|  | Unthinned, scores ≥ 9 | 700 / 700 BP | 1250 CE | 534 / 534 BP | **1416 CE** | 309 / 309 BP | 1641 CE | 76 | Scores9 |
|  | Unthinned, scores < 9 | 49 /  49 BP | 1901 CE | -212 / 212 AD | **2162 CE** | -434 / 434 AD | 2290 CE | 67 | Scoresbelow9 |
| **North-West** | Unthinned, all | 108 / 108 BP | 1842 CE | -124 / 124 AD | **2074 CE** | -340 / 340 AD | 2290 CE | 74 | NWunthinned |
|  | Thinned,  all | 97 /  97 BP | 1853 CE | -207 / 207 AD | **2157 CE** | -1355 / 1355 AD | 3305 CE | 45 | NWthinned |
|  | Unthinned, scores ≥ 9 | 710 / 710 BP | 1240 CE | 530 / 530 BP | **1420 CE** | 293 / 293 BP | 1657 CE | 41 | NW9 |
|  | Unthinned, scores < 9 | 39 /  39 BP | 1911 CE | -258 / -258 AD | **2208 CE** | -511 /  511 AD | 2461 CE | 33 | NWbelow9 |
| **Maine** | Unthinned, all | 80 /  80 BP | 1870 CE | -175 /  175 AD | **2125 CE** | -409 /  409 AD | 2359 CE | 59 | MEunthinned |
|  | Thinned,  All | 89 /  89 BP | 1861 CE | -235 /  235 AD | **2185 CE** | -1397 /  1397 AD | 3347 CE | 34 | MEthinned |
|  | Unthinned, scores ≥ 9 | 711 /  711 BP | 1239 CE | 531 /  531 BP | **1419 CE** | 283 /  283 BP | 1667 CE | 36 | ME9 |
|  | Unthinned, scores < 9 | -7 /  7 AD | 1957 CE | -397 /  397 AD | **2347 CE** | -694 /  694 AD | 2644 AD | 21 | MEbelow9 |
| **North-East** | Unthinned, all | 475 /  475 BP | 1475 CE | 228 / 228 BP | **1722 CE** | -10/  10 AD | 1960 CE | 69 | NEunthinned |
|  | Thinned,  all | 245 / 245 BP | 1705 CE | -69 /  69 AD | **2019 CE** | -354 /  354 AD | 2304 CE | 34 | NEthinned |
|  | Unthinned, scores ≥ 9 | -212 / 212 AD | 2162 CE | -498 /  498 AD | **2448 CE** | -767 / 767 AD | 2717 CE | 26 | NE9 |
|  | Unthinned, scores < 9 | 1430 / 1430 BP | 520 CE | 1103 / 1103 BP | **847**  **CE** | 709 / 709 BP | 1241 CE | 43 | NEbelow9 |
|  | Direct Dates | -1484 / 1484 AD | 3434 CE | -1885 / 1885 AD | **3835**  **CE** | -2281 AD / 2281 AD | 4231 CE | 9 | Directdates |

Our median results for extinction timing estimates, both thinned and unthinned, largely fall post 1950 CE, with 95% bands typically extending from the 19^th^ century to beyond the 21^st^ century. Thes values are in contrast with the last confirmed Great Auk, killed in 1844 CE (e.g., 106 years BP) (Supplemental Figure 2). Estimates from the thinned dataset produce similar median estimates of extinction but much wider distributions extending into the future, consistent with the use of a smaller dataset. Further, unthinned datasets produce more recent extinction estimates because of the inherently tighter clustering of dates that have smaller gaps between observations in the fossil record.

GRIWM has been known to produce anomalously recent extinction dates and confidence limits that extend into the future for several taxa (i.e., are negative model outputs), notably for those that are either very poorly sampled with a small number of radiocarbon dates spaced widely in time and/or for species with Late Holocene extinctions close to the present with radiocarbon dates that have large uncertainty ranges [21;22;23;24]. Such wide uncertainties are also expected when considering radiocarbon dates from marine samples, given issues associated with offsets and reservoir corrections [25]. While such uncertainties are reasonable on the scale of events across millennia or centuries, such as for Late Quaternary megafauna that went extinct at the end of the Pleistocene, they can complicate understanding of historical events where decadal resolution is important [26].

Supplemental Figure 2. A visual comparison of GRIWM model extinction timing results of the thinned and unthinned datasets (including dates of all quality scores). This highlights that thinning the datasets did not change the extinction estimates significantly, though have larger ranges likely due to having a smaller dataset with larger gaps between dates that still contain dates close in time to the present. Year is reported in CE.

*(e) Radiocarbon-based extinction modeling with CRIWM*

A recent update to the GRIWM approach was published in 2024: the Calibration-Resampled Inverse-Weighted McInerny Method (CRIWM) as implemented in the R package *Rextinct* [27]. This method was updated to better incorporate the probability density functions of radiocarbon calibration and does not assume normal chronometric errors. Differences in extinction estimates between GRIWM and CRIWM for a series of 29 training datasets were found to be on the order of decades [27]. Unfortunately, this model cannot accommodate marine date calibration with multiple different marine reservoirs corrections, which is exactly what is needed for a widely distributed marine organisms such as the Great Auk (see [28], a recently published paper in 2025, for an extended discussion of this issue as it related to historical whale samples; Saltré and Herrando-Pérez, personal communication). Because CRIWM relies on rcarbon::calibrate() for radiocarbon calibration (via the *rcarbon* package), it cannot utilize multiple custom calibration curves simultaneously as would be inherently necessary for our dataset that includes marine dates across very different areas of the Atlantic that have very different marine reservoir offsets. The R package *Rextinct* itself would need to be modified; this package is neither designed to handle this nor can currently handle this at this time, though it is certainly a clear future priority for radiocarbon-based extinction model development. We suggest that this dataset be revisited following development of such tools and additional radiocarbon dating of Great Auk remains across their range.

To implement CRIWM for our Great Auk dataset restricts us to terrestrial radiocarbon dates and forces us to discard dates requiring marine calibration and reservoir correction. We stress that this approach is inadvisable because Great Auks are inherently marine organisms and are found in coastal and insular contexts, including archaeological shell deposits. By not being able to use marine dates, we remove some of the most accurate contexts and importantly, remove bones from Great Auks themselves, thus removing the highest quality and only direct dates from the analysis of extinction timing; it also requires removal of the most recent date in the entire dataset and produces a large gap in dates towards the oldest range of occurrences (Supplemental Figure 1). Great Auks and other highly mobile marine organisms, such as whales, have remains that span multiple different carbon mixing reservoirs and thus require the use of different local reservoir corrections that vary on the scale of centuries. Van den Hurk et al [28] also argue that GRIWM is also well suited for the use of calibrated marine dates because it considers radiocarbon dates as normally distributed and marine calibrated dates produce probability density functions that are more unimodal.

However, to provide a point of contrast, we implemented CRIWM in the R package *Rextinct* [27] for the entire geographic range, Northwest Atlantic, and Northeast Atlantic population groups and focused on only terrestrial unthinned radiocarbon dates, thereby excluding all directly dated Great Auk material. All CRIWM estimates of extinction are earlier than those produced by GRIWM (Supplemental Table 2). For example, we found a median extinction date of 1718 CE for the entire range, a date preceding the known last capture of a Great Auk at 1844 CE (Supplemental Table 2).

Supplemental Table 2. Comparison of GRIWM and CRIWM extinction timing results. All datasets included are unthinned and include all dates of all quality scores. CRIWM datasets contain only terrestrial dates due to the limitations of the *Rextinct* R package as it is currently coded by developers [27].

| **GRIWM/ CRIWM** | **Range** | **Lower (output)** | **Lower (CE)** | **Median**  **(output)** | **Median (CE)** | **Upper (output)** | **Upper (CE)** | **Sample Size** | **Filename (.csv)** |
| --- | --- | --- | --- | --- | --- | --- | --- | --- | --- |
| GRIWM | Entire Range | 124 / 124 BP | 1826 CE | -87 /  87 AD | **2037 CE** | -284 / 284 AD | 2234 CE | 143 | GRIWMEntireRange |
| CRIWM | Entire Range | 386 / 386 BP | 1564 CE | 232 /  232 BP | **1718 CE** | 87 /  87 BP | 1863 CE | 108 | CRIWMall |
| GRIWM | North- West | 108 / 108 BP | 1842 CE | -124 / 124 AD | **2074 CE** | -340 / 340 AD | 2290 CE | 74 | NWunthinned |
| CRIWM | North-West | 391 / 391 BP | 1559 CE | 228 / 228 BP | **1722 CE** | 74 /  74 BP | 1876 CE | 57 | CRIWMnorthwest |
| GRIWM | North-East | 475 / 475 BP | 1475 CE | 228 / 228 BP | **1722 CE** | -10 /  10 AD | 1960 CE | 69 | NEunthinned |
| CRIWM | North-East | 1887 / 1887 BP | 63 CE | 1237 / 1237 BP | **713 CE** | 743 / 743 BP | 1207 CE | 51 | CRIWMnortheast |

2. Sighting-based methods & outputs

*(a) Historical sighting database curation*

We assembled a historical sighting database by searching the Global Biodiversity Information Facility (GBIF) [29] and literature including historical whaling archives, testimonies, and diaries in print and online. We used Google Translate to translate historical documents from other languages (Norwegian, Icelandic, Faroese, Latin, Spanish, French, Danish) into English, combed the literature, and contacted collection managers of historical archives. We searched for any mentions of Great Auk under its many alternative names and spelling variations (see “Great Auk Names supplemental file” and Appendix for the complete list and references). For each sighting, we recorded locality, latitude, longitude, date (CE), reporter, and reference details. We used GEOLocate [8] to determine geographic coordinates and assigned quality scores (1-3) to all records based on their credibility: 1, specimen collected at known date; 2, confirmed sighting without associated physical specimen; 3, unconfirmed sighting (Supplemental Figure 3).

For instance, this description would merit a confirmed sighting with a score of two due to its accurate details:

*Some of these birds are as large as geese, being black and white with a beak like a*	*crow’s. They are always in the water, not being able to fly in the air, inasmuch as they*	*have only small wings about the size of one’s hand, with which however they move as*	*quickly along the water as other birds fly through the air. And these birds are so fat that*	*it is marvelous…* [30].

If there were more than one sighting per year, we omitted duplicate sightings based on reliability, keeping the highest-scoring date.

Supplemental Figure 3. All sighting data subset by quality. Year is reported in CE. Note that confirmed specimens are biased towards the end of the Great Auk’s lifetime given the timeline of development of natural history collections. As expected, numerous low-quality/unconfirmed sightings are clustered towards the end of the observation period, when public interest increased following reports of species demise [31;32].

*(d) Sighting-based extinction modeling*

We analysed historical sighting data using a Bayesian extinction timing model produced by Solow & Beet [32] which accounts for sighting quality, with specimen-based and confirmed sightings having a greater impact on the extinction date estimate than unconfirmed sightings. Whereas early sighting-based models assume all sightings are valid and do not account for uncertainty (e.g., the optimal linear estimator approach [33;34], we implemented Solow and Beet’s “Model 2”, as it includes mixed-certainty sighting data to model extinction timing more robustly, by considering specimen-based, confirmed, and unconfirmed sightings within the same Poisson process such that sightings of varying validity can co-occur before the true extinction date. This model has been applied to the study of several historically extinct birds and mammals [35;31;36]. Our certain sightings are valid (being physical preserved specimens) and the uncertain sightings are likely a combination of true and false observations.

We input our data in 2 columns (Year, Sightings) with year in CE and “Sightings” representing the quality score of the sighting, with 1 being a specimen collected at known date; 2 being an confirmed sighting without an associated physical specimen; and 3, an unconfirmed sighting (see Table 3). We conducted compared estimates across different regions and included date ranges when given (see Historical Sighting Dataset on GitHub). To determine temporal patterns between specimen-based and unconfirmed sightings, we assessed model outputs separately across the entire geographic range. We conducted combined analyses of specimen-based and confirmed sightings (scores 1+2) and confirmed and unconfirmed sightings (scores 2+3); it was not possible to analyse each score separately due to the low sample size of dates. We also re-ran the model to estimate extinction using the earliest, median, and latest dates when reports provided a range of possible dates. We estimated extinction timing for the species’ entire range and for designated populations with sufficient sample sizes: the Northwest Atlantic and the Northeast Atlantic.

We used an R script modified from [31;35] to implement Solow & Beet’s “Model 2” (see GitHub). We chose the year 1870 CE to represent the end of the observation period, as this is the last sighting (unconfirmed) in our dataset, consistent with the approach taken by Solow & Beet themselves when modeling the extinction of the Ivory-Billed Woodpecker [32]. We selected a uniform prior (posteriorPrior = "Unif"), meaning possible extinction dates between the last certain sighting (1844) and last unconfirmed sighting (1870) are considered equally likely in the model. Following [31;35], we set the tuning parameter gamma = 6 and the model was run in increments of 0.001 [35;36].

This R script employs Solow and Beet’s (2014) Bayesian extinction estimation equations (as modified from Solow et al. (2012)) [32;37] to estimate extinction date (τE) of the Great Auk. As noted above, this approach considers different types of evidence (e.g., museum specimen vs. eye witness observation) and certainty levels independently [35;36;38;39].

As documented directly in [31;35;36] and reproduced here, Solow & Beet (2014)’s general equation (Equation 1) is:

$$p(E|t)= \frac{p\left( t | E \right)p(E)}{p(t|E)p\left( E \right)+p(t|Ē)(1-p\left( E \right))}$$

Where:

*E* is the event the species went extinct in a given year, Ē is the event when the species did not go extinct in a given year (i.e., it persists to exist), and *t* is the dataset consisting of all sightings for that species (t = t_1_ < t_2_ < … < t_n_) [31;35;36].

We chose to use “Model 2” because it is best suited for datasets in which uncertain sightings are more likely to be non-accurate (Kodikara et al 2018). Similar to Burgio et al (2021)’s assessment of uncertain non-accurate sightings for the Carolina Parakeet, the Great Auk could have been mistakenly identified d for a variety of reasons, such as the Great Auk looking similar to other birds, public familiarity, and the desire to profit from Great Auk feathers as they became increasingly rare during the feather trade.

As noted in [31;35;36] and reproduced here, Equation 1 is modified into Model 2 as Equation 9 of Solow & Beet (2014).

$$p\left( t | \tau_{E} \right)=p(t_{c}|\tau_{E})p(t_{u}|\tau_{E})$$

Where t_c_ is the set of certain sighting times and t_u_ is the set of uncertain sighting times [31;35;36].

To avoid bias, and following [31;35], we assume an uninformative prior where it is equally likely that the species went extinct or the species persisted:

$$P\left( E \right)= P(Ē))=0.5$$

With a posterior probability of:

$$P\left( E | t \right)=\frac{P\left( t | E \right)}{P\left( t | E \right)+ P\left( t | Ē \right)}$$

Using this range of observations through 1870 CE, we generated a τ_E_ of 1860 CE. Purely for exploratory purposes, to see if there is a difference if we choose a different test year that was closer to the present as suggested by our GRIWM median extinction timing estimates that extend past the 21^st^ century, we changed T from 1870 CE and 2000 CE. The result is a difference in extinction estimate was shifted by ~20 years, still remaining prior to 1900 CE (see Supplemental Figures 4 & 5). However, we stress that there are no sightings (of any quality) following 1870 CE, and thus primarily report our extinction estimates based on an observation period of 1870 CE, following the convention of the authors of this modelling approach.

Supplemental Figure 4. Re-run of the Solow and Beet sighting Model 2 using a different test year of 2000 CE but using the same sighting data for the Northwest Atlantic Great Auk population. The extinction estimate is 1880 CE (95% CI: 1848-1994 CE, *p*(*t*|τ*_E_*) = 7.60×10^137^) compared to 1860 CE (95% CI: 1825-1867 CE, *p*(*t*|τ*_E_*) = 2.21×10^56^) (Figure 3) when a test year of 1870 was chosen as that is the last known date of the observation period. As described in [35], this figure displays the posterior probability of τ_E_, scaled by the area below the likelihood curve.

Supplemental Figure 5. Re-run of the Solow and Beet sighting Model 2 using a different test year of 2000 but using the same sighting data for the Northeast Atlantic Great Auk population. The extinction estimate is 1880 CE (95% CI: 1848-1994 CE, *p*(*t*|τ*_E_*) = 7.60×10^137^) compared to 1851 CE (95% CI: 1848-1867 CE, *p*(*t*|τ*_E_*) = 2.00×10^57^) (Figure 3) when a test year of 1870 was chosen as that is the last known date of the observation period. As described in [35], this figure displays the posterior probability of τ_E_, scaled by the area below the likelihood curve.

Supplemental Table 3. All extinction timing outputs from the sighting-based Solow and Beet Model 2. For the entire range, we ran 4 different runs to include date ranges (in which a historical record mentioned a range of dates possible for one sighting). As this seemed to have no effect on extinction estimates, we did not do this for the specific regions. We modeled all sighting dates for the Northwest and Northeast Atlantic regions. We also ran expert and confirmed sightings together in one run and confirmed and unconfirmed sightings together in another run to compare estimates between datasets of differing quality.

| **Region** | **Run** | **Year (CE)** | **Integrand** | **Sample Size** | **Filename (.csv)** |
| --- | --- | --- | --- | --- | --- |
| **Entire Range** | All (without date ranges) | **1859.71** | **2.71715E+128** | 96 | allsightings |
|  | Including earliest date of range | **1859.71** | **3.075567E+133** | 99 | earliestofdaterange |
|  | Including middle date of range | **1859.71** | **2.169897E+130** | 97 | middleofdaterange |
|  | Including last date of range | **1859.71** | **9.427908E+140** | 103 | lastofdaterange |
| **North-West** | All | **1859.71** | **2.206326E+56** | 49 | NWsightingsall |
| **North-East** | All | **1850.74** | **2.001407E+57** | 55 | NEsightingsall |
| **Other** | Expert and confirmed sightings | **1848.48** | **8.148457E+111** | 87 | expertandconfirmedsightings |
|  | Confirmed and unconfirmed sightings | **1859.71** | **6.965204E+111** | 79 | confirmedandunconfirmedsightings |

References

[1] CARD [Internet]. [cited 2024 Mar 27]. Available from: <https://www.canadianarchaeology.ca/>

[2] McClennen M, Jenkins J, Uhen M. Paleobiology Database [Internet]. 2017 [cited 2024 Mar 27]. Available from: [https://www.gbif.org/dataset/bb5b30b4–827e–4d5e–a86a–825d65cb6583](https://www.gbif.org/dataset/bb5b30b4-827e-4d5e-a86a-825d65cb6583)

[3] Mead J, Meltzer D. North American Late Quaternary extinctions and the radiocarbon record. In: Quaternary Extinctions: a prehistoric revolution. Tucson: University of Arizona Press; 1984. p. 440–50.

[4] Barnosky AD, Lindsey EL. Timing of Quaternary megafaunal extinction in South America in relation to human arrival and climate change. Quaternary International. 2010 Apr;217(1–2):10–29.

[5] Reimer PJ, Austin WEN, Bard E, Bayliss A, Blackwell PG, Bronk Ramsey C, et al. The IntCal20 Northern Hemisphere Radiocarbon Age Calibration Curve (0–55 cal kBP). Radiocarbon. 2020 Aug;62(4):725–57.

[6] Bronk Ramsey C. Radiocarbon Calibration and Analysis of Stratigraphy: The OxCal Program. Radiocarbon. 1995;37(2):425–30.

[7] Heaton TJ, Köhler P, Butzin M, Bard E, Reimer RW, Austin WEN, et al. Marine20—The Marine Radiocarbon Age Calibration Curve (0–55,000 cal BP). Radiocarbon. 2020;62(4):779–820. doi:10.1017/RDC.2020.68

[8] Rios N, Bart H. GEOLocate. Belle Chasse, LA: Tulane University Museum of Natural History.; 2010.

[9] Fernández-López de Pablo, J., Gutiérrez-Roig, M., Gómez-Puche, M. *et al.* Palaeodemographic modelling supports a population bottleneck during the Pleistocene-Holocene transition in Iberia. *Nat Commun* **10**, 1872 (2019). https://doi.org/10.1038/s41467-019-09833-3

[10] Spiess, A., & Lewis, R. A. (2001). The Turner Farm fauna: 5000 years of

hunting and fishing in Penobscot Bay, Maine. Maine State Museum: Maine

Historic Preservation Commission: Maine Archaeological Society.

[11] Asle Bruen Olsen. Kotedalen - en boplass gjennom 5000 år. 1: Fangstbosetning og tidlig fordbruk i Vestnorsk Steinalder: Nye funn og nye perspektiver. Bergen: Historical Museum, University of Bergen; 1992.

[12] Dockrill, S. J., & Bond, J. M. (2009). Sustainability and resilience in prehistoric North Atlantic britain: The importance of a mixed paleoeconomic system. Journal of the North Atlantic, 2, 33–50. <https://doi.org/10.3721/037.002.0105>

[13] Cox, S. L. (2021). Goddard: A Prehistoric Village Site on Blue Hill Bay, Maine. Maine Historic Preservation Commission.

[14] Hamilton, N. D. (1985). Maritime Adaptation in Western Maine: The Great Diamond Island Site [PhD]. University of Pittsburgh.

[15] Watson, J. E. (2019). Human Interactions with the Late Holocene Landscape on Martha’s

Vineyard [PhD]. University of Albany, State University of New York.

[16] Fischer, A., Gotfredsen, A. B., Meadows, J., Pedersen, L., & Stafford, M. (2021). The rødhals kitchen midden – marine adaptations at the end of the Mesolithic World. Journal of Archaeological Science: Reports, 39. <https://doi.org/10.1016/j.jasrep.2021.103102>

[17] James, H., & McCullagh, R. (2003). Excavations at Hornish Point. Scottish Archaeological Internet Reports, 72-103.

[18] Bradshaw CJA, Cooper A, Turney CSM, Brook BW. Robust estimates of extinction time in the geological record. Quaternary Science Reviews. 2012 Feb;33:14–9.

[19] F. Robin O’Keefe et al., Pre–Younger Dryas megafaunal extirpation at Rancho La Brea linked to fire-driven state shift.*Science***381**,eabo3594(2023).DOI:[10.1126/science.abo3594](https://doi.org/10.1126/science.abo3594)

[20] R Core Team. R: A Language and Environment for Statistical Computing. R Foundation for Statistical Computing. Vienna, Austria. 2024. Available from: [https://www.R–project.org/](https://www.r-project.org/).

[21] A.D. Barnosky, E.L. Lindsey, N.A. Villavicencio, E. Bostelmann, E.A. Hadly, J. Wanket, & C.R. Marshall. Variable impact of late-Quaternary megafaunal extinction in causing ecological state shifts in North and South America, *Proc. Natl. Acad. Sci. U.S.A.* 113 (4) 856-861, <https://doi.org/10.1073/pnas.1505295112> (2015).

[22] Cooke SB, Mychajliw AM, Southon J, MacPhee RDE. The extinction of *Xenothrix mcgregori*, Jamaica’s last monkey. Journal of Mammalogy. 2017 Aug 1;98(4):937–49. doi:[10.1093/jmammal/gyw165](https://doi.org/10.1093/jmammal/gyw165)

[23] Francisco J. Prevosti, Mariano A. Ramírez, Mauro Schiaffini, Fabiana Martin, Daniel E. Udrizar Sauthier, Marcelo Carrera, Claudio Sillero-Zubiri, Ulyses F. J. Pardiñas, Extinctions in near time: new radiocarbon dates point to a very recent disappearance of the South American fox Dusicyon avus (Carnivora: Canidae), Biological Journal of the Linnean Society, Volume 116, Issue 3, November 2015, Pages 704–720, <https://doi.org/10.1111/bij.12625>

[24] **Saltré, F., Brook, B. W., Rodríguez-Rey, M., Cooper, A., Johnson, C. N., Turney, C. S. M., & Bradshaw, C. J. A. (2015).** Uncertainties in dating constrain model choice for inferring extinction time from fossil records. **Quaternary Science Reviews, 112,** 128–137. https://doi.org/10.1016/j.quascirev.2015.01.022

[25] **Alves, E. Q., Macario, K. D., Urrutia-Fucugauchi, J., Cardoso, R. P., & Bronk Ramsey, C. (2019).** Accounting for the marine reservoir effect in radiocarbon calibration. **Quaternary Science Reviews, 209,** 129–138. https://doi.org/10.1016/j.quascirev.2019.02.013

[26] Mychajliw, A. M., Adams, A. J., Brown, K. C., Campbell, B. T., Hardesty-Moore, M., Welch, Z. S., Page, H. M., Southon, J. R., Cooper, S. D., & Alagona, P. S. (2024). Coupled social and ecological change drove the historical extinction of the California grizzly bear (*Ursus arctos californicus*). *Proceedings. Biological sciences*, *291*(2014), 20230921. https://doi.org/10.1098/rspb.2023.0921

[27] Herrando–Pérez S, Saltré F. Estimating extinction time using radiocarbon dates. Quaternary Geochronology. 2024 Jan;79:101489.

[28] Van Den Hurk Y, Sikström F, Amkreutz L, Bakker H, Buss DL, Ersmark E, et al. Dating the first historic extirpation of a whale species: The demise of the grey whale (*Eschrichtius robustus*) in the eastern North Atlantic. Quaternary Science Reviews. 2025 Dec;369:109583. doi:[10.1016/j.quascirev.2025.109583](https://doi.org/10.1016/j.quascirev.2025.109583)

[29] What is GBIF? [Internet]. [cited 2024 Mar 27]. Available from: [https://www.gbif.org/what–is–gbif](https://www.gbif.org/what-is-gbif)

[30] The First Relation of Jaques Carthier of S. Malo, 1534 [Internet]. Wisconsin Historical Society; 1534. (Original Narratives of Early American History). Available from: [http://www.americanjourneys.org/aj–026/](http://www.americanjourneys.org/aj-026/)

[31] Burgio KR, Carlson CJ, Bond AL, Rubega MA, Tingley MW. The two extinctions of the Carolina Parakeet *Conuropsis carolinensis*. Bird Conservation International. 2021 Jul 21;1–8.

[32] Solow AR, Beet AR. On Uncertain Sightings and Inference about Extinction. Conservation Biology. 2014;28(4):1119–1123. <http://www.jstor.org/stable/24480090>

[33] Clements, C.F., Worsfold, N.T., Warren, P.H., Collen, B., Clark, N., Blackburn, T.M. and Petchey, O.L. (2013), Experimentally testing the accuracy of an extinction estimator: Solow's optimal linear estimation model. J Anim Ecol, 82: 345-354. <https://doi.org/10.1111/1365-2656.12005>

[34] Roberts, D., Solow, A. When did the dodo become extinct?. *Nature* **426**, 245 (2003). <https://doi.org/10.1038/426245a>

[35] Bond, A.L., Carlson, C.J. & Burgio, K.R. Local extinctions of insular avifauna on the most remote inhabited island in the world. *J Ornithol* **160**, 49–60 (2019). https://doi.org/10.1007/s10336-018-1590-8

[36] Carlson CJ, Bond AL, Burgio KR. Estimating the extinction date of the thylacine with mixed certainty data. Conservation Biology. 2018 Apr;32(2):477–83.

[37] Solow, A., Smith, W., Burgman, M., Rout, T., Wintle, B. and Roberts, D. (2012), Uncertain Sightings and the Extinction of the Ivory-Billed Woodpecker. Conservation Biology, 26: 180-184. <https://doi.org/10.1111/j.1523-1739.2011.01743.x>

[38] **Boakes, E. H., Collen, B., Rout, T. M., & others. (2015). Inferring species extinction: the use of sighting records.** Methods in Ecology and Evolution, **6**(6), 678–687.
<https://doi.org/10.1111/2041-210X.12365>

[39] **Kodikara, S., Demirhan, H., & Stone, L. (2018). Inferring about the extinction of a species using certain and uncertain sightings.** Journal of Theoretical Biology, **442,** 98–109. https://doi.org/10.1016/j.jtbi.2018.01.015

Appendix

1. **OxCal Code**

Plot()

{

Sequence("Turner Farm Phase 1")

{

Boundary("Start");

Phase("Stratum 4")

{

R_Date(875,70);

R_Date(1200,100);

R_Date(1955,50);

R_Date(2105,75);

R_Date(2275,130);

R_Date(3185,65);

First();

Last();

Span();

};

Boundary("End");

};

};

Plot()

{

Combine("Turner Farm Marine Stratum 4")

{

R_Date(2530,65);

R_Date(2575,75);

// Delta_R values checked for Marine20

Curve("Marine20","marine20.14c");

Delta_R(72,61);

};

};

Plot()

{

Sequence("Turner Farm Phase 2")

{

Boundary("Start");

Phase("Stratum 3 Terrestrial")

{

R_Date(3470,60);

R_Date(3480,75);

R_Date(3515,80);

R_Date(3610,90);

R_Date(3630,85);

R_Date(3650,75);

R_Date(3700,85);

R_Date(3710,80);

R_Date(3825,65);

R_Date(3855,75);

R_Date(4020,80);

Curve("IntCal20","intcal20.14c");

First();

Last();

Span();

};

Boundary("End");

};

};

Plot()

{

Combine("Turner Farm Marine Stratum 3")

{

R_Date(3270,75);

R_Date(3450,75);

// Delta_R values checked for Marine20

Curve("Marine20","marine20.14c");

Delta_R(72,61);

};

};

Plot()

{

Sequence("Turner Farm Phase 3")

{

Boundary("Start");

Phase("Stratum 2 Terrestrial")

{

R_Date(2705,60);

R_Date(3115,65);

R_Date(3480,90);

R_Date(3920,25);

R_Date(4005,55);

R_Date(4050,220);

R_Date(4390,55);

R_Date(4410,80);

R_Date(4555,95);

Curve("IntCal20","intcal20.14c");

First();

Last();

Span();

};

Boundary("End");

};

};

Plot()

{

Sequence("Turner Farm Phase 3")

{

Boundary("Start");

Phase("Stratum 2 Marine")

{

R_Date(3665,55);

R_Date(3710,60);

R_Date(3745,75);

R_Date(3785,75);

R_Date(4135,85);

First();

Last();

Span();

};

Boundary("End");

};

};

// Delta_R values checked for Marine20

Curve("Marine20","marine20.14c");Delta_R(72,61);

Plot()

{

Sequence("Køtedalen Phase 2")

{

Boundary("Start");

Phase("2 Terrestrial")

{

R_Date(7230,100);

R_Date(7330,110);

R_Date(7440,110);

R_Date(7610,90);

R_Date(8530,135);

Curve("IntCal20","intcal20.14c");

First();

Last();

Span();

};

Boundary("End");

};

};

Plot()

{

Sequence("Køtedalen Phase 12")

{

Boundary("Start");

Phase("12 Terrestrial")

{

R_Date(4900,100);

R_Date(5010,50);

R_Date(5010,80);

R_Date(5030,80);

R_Date(5050,70);

R_Date(5100,60);

R_Date(5160,120);

Curve("IntCal20","intcal20.14c");

First();

Last();

Span();

};

Boundary("End");

};

};

Plot()

{

Sequence("Køtedalen Phase 13")

{

Boundary("Start");

Phase("13 Terrestrial")

{

R_Date(4860,60);

R_Date(4920,60);

R_Date(4960,80);

Curve("IntCal20","intcal20.14c");

First();

Last();

Span();

};

Boundary("End");

};

};

Plot()

{

Sequence("Køtedalen Phase 14")

{

Boundary("Start");

Phase("14 Terrestrial")

{

R_Date(4560,110);

R_Date(4610,80);

R_Date(4640,60);

R_Date(4650,90);

R_Date(4660,120);

R_Date(4670,90);

R_Date(4690,70);

R_Date(4690,90);

R_Date(4760,100);

R_Date(4880,80);

Curve("IntCal20","intcal20.14c");

First();

Last();

Span();

};

Boundary("End");

};

};

Plot()

{

Sequence("Køtedalen Phase 15")

{

Boundary("Start");

Phase("15 Terrestrial")

{

R_Date(4280,60);

R_Date(4340,50);

R_Date(4340,90);

R_Date(4410,50);

R_Date(4410,90);

R_Date(4430,50);

R_Date(4450,50);

R_Date(4490,70);

R_Date(4580,80);

Curve("IntCal20","intcal20.14c");

First();

Last();

Span();

};

Boundary("End");

};

};

Plot()

{

Combine("Tofts Ness Phase 3")

{

R_Date(3270,50);

R_Date(3380,70);

R_Date(3390,60);

Curve("IntCal20","intcal20.14c");

};

};

Plot()

{

Combine("Tofts Ness Phase 2")

{

R_Date(3550,90);

R_Date(3650,50);

R_Date(4160,90);

R_Date(4230,90);

Curve("IntCal20","intcal20.14c");

};

};

Plot()

{

Combine("Tofts Ness Phase 1")

{

R_Date(4430,70);

R_Date(4480,70);

Curve("IntCal20","intcal20.14c");

};

};

Plot()

{

Combine("Diamond B IIIb Terrestrial")

{

R_Date(1520,55);

R_Date(1600,95);

Curve("IntCal20","intcal20.14c");

};

};

Plot()

{

Combine("Frisby Butler Stratum 2 Terrestrial")

{

R_Date(2060,15);

R_Date(2085,15);

R_Date(2095,15);

Curve("IntCal20","intcal20.14c");

};

};

Plot()

{

Combine("Frisby Butler Stratum 3 Terrestrial")

{

R_Date(2165,15);

R_Date(2100,20);

Curve("IntCal20","intcal20.14c");

};

};

Plot()

{

Sequence("Sejerø")

{

Boundary("Start");

Phase("Entire")

{

R_Date(4705,20);

R_Date(4980,20);

R_Date(5015,20);

R_Date(5020,20);

R_Date(5195,20);

Curve("IntCal20","intcal20.14c");

First();

Last();

Span();

};

Boundary("End");

};

};

2. **Great Auk Names**

| **Name(s)** | **Locality** | **Number of sightings** | **Reference** |
| --- | --- | --- | --- |
| Abâktoo’e-âk | Maine | 0 | Silas Tertius Rand. A first reading book in the Micmac language: Comprising the Micmac numerals, and the names of the different kinds of beasts, birds, fishes, trees, &c. of the maritime provinces of Canada. Also, some of the Indian names of places, and many familiar words and phrases, translated literally into English. Halifax: Nova Scotia Printing Company; 1875. |
| Alca impennis | Entire range | 1 | John Fleming. Gleanings of natural history during a voyage along the coast of Scotland in 1821. Edinburgh Philosophical Journal. 1824;10:95–101. |
| Alca borealis | Sweden | 0 | Thomas T. Forster. A synoptical catalogue of British birds; intended to identify the species mentioned by different names in several catalogues already extant [Internet]. London: Printed by and for Nichols, son, and Bentley; 1817. Available from: https://doi.org/10.5962/bhl.title.13346 |
| Alk(a) | Scottish isles | 0 | Wilhelm Blasius. Der Riesenalk. Naumann, Naturgeschichte der Vögel Mitteleuropas. 1903;7. |
| An erbhoil/An Gearbhul/  An Gearra-bhul | United Kingdom | 0 | Wilhelm Blasius. Der Riesenalk. Naumann, Naturgeschichte der Vögel Mitteleuropas. 1903;7.; Gray R. Birds of the West of Scotland Including The Outer Hebrides. 1st ed. Glasgow: Thomas Murray; 1871. |
| Anglema(a)ge/Angledemager/  Anglemager | Denmark | 0 | Wilhelm Blasius. Der Riesenalk. Naumann, Naturgeschichte der Vögel Mitteleuropas. 1903;7.; Victor Fatio. Histoire naturelle des oiseaux de la Suisse [Internet]. Vol. 1. Genève et Bale: Musée de Zoologie Lausanne Bibliothèque; 1899. Available from: https://doi.org/10.5962/bhl.title.124601 |
| Anser Magellanicus, seu Pinguini | Denmark | 1 | Ole Worm. Museum Wormianum, seu, Historia rerum rariorum : tam naturalium, quam artificialium, tam domesticarum, quam exoticarum, quae Hafniae Danorum in aedibus authoris servantur [Internet]. 1st ed. Copenhagen: Lugduni Batavorum : Apud Iohannem Elsevirium; 1655. Available from: https://archive.org/details/gri_museumwormia00worm/page/n11/mode/2up |
| Ap(p)onar/t | Newfoundland | 1 | André Thevet. Les singularitez de la France Antarctique, autrement nommée Amerique, & de plusieurs Terres & Isles decouvertes de nostre temps. 2nd ed. Antwerp: Christopher Plantin; 1558. |
| Aponas | Newfoundland | 0 | Wilhelm Blasius. Der Riesenalk. Naumann, Naturgeschichte der Vögel Mitteleuropas. 1903;7. |
| Aporath | Newfoundland | 3 | Henry S. Burrage. Early English and French Voyages. American Historical Association; 1906. 13 p.; The First Relation of Jaques Carthier of S. Malo, 1534 [Internet]. Wisconsin Historical Society; 1534. (Original Narratives of Early American History). Available from: http://www.americanjourneys.org/aj-026/ |
| Apponath/Apponatz | Newfoundland | 1 | [The First Relation of Jaques Carthier of S. Malo, 1534 [Internet]. Wisconsin Historical Society; 1534. (Original Narratives of Early American History). Available from: http://www.americanjourneys.org/aj-026/](http://www.americanjourneys.org/aj-026/) |
| Auk | Scottish isles | 0 | Wilhelm Blasius. Der Riesenalk. Naumann, Naturgeschichte der Vögel Mitteleuropas. 1903;7. |
| Avis-Gare | Scotland | 0 | Robert Sibbald. De Animalibus Scotiæ. Vol. 2. Edinburgh; 1684. 22 p. |
| Avis Garfahl | Sweden | 0 | Carl von Linné. Fauna Svecica, sistens animalia Sveciae regni : quadrupedia, aves, amphibia, pisces, insecta, vermes, distributa per classes & ordines, genera & species, cum differentiis specierum, synonymis autorum, nominibus incolarum, locis habitationum, descriptionibus insectorum [Internet]. Stockholm: Lugduni Batavorum; 1746. Available from: https://doi.org/10.5962/bhl.title.63897 |
| Barricardières | France | 0 | Wilhelm Blasius. Der Riesenalk. Naumann, Naturgeschichte der Vögel Mitteleuropas. 1903;7. |
| Binocle | North America | 0 | Wilhelm Blasius. Der Riesenalk. Naumann, Naturgeschichte der Vögel Mitteleuropas. 1903;7. |
| Bonnivochil/Bishop-fowl | Scotland | 0 | Wilhelm Blasius. Der Riesenalk. Naumann, Naturgeschichte der Vögel Mitteleuropas. 1903;7. |
| Brillefugl | Denmark | 0 | [Grieve S. The great auk, or garefowl (alca impennis, Linn.): Its history, archaeology, and remains. Edinburgh, Scotland: Grange Publishing Works; 1885; Thomas Pennant. Arctic Zoology [Internet]. Vol. 2. London: Henry Hughs; 1785. Available from: https://doi.org/10.5962/bhl.title.16548](https://doi.org/10.5962/bhl.title.16548) |
| Brillenalk | Denmark | 0 | Grieve S. The great auk, or garefowl (alca impennis, Linn.): Its history, archaeology, and remains. Edinburgh, Scotland: Grange Publishing Works; 1885. |
| Bunnabhuächaille | Scotland | 0 | Martin M. A description of the Western Islands of Scotland. 2nd ed. London; 1703. |
| Chenalopex impennis | United Kingdom | 0 | Preyer W. Ueber Plautus impennis (Alca impennis L.). A. Emmerling; 1862. |
| Der Riesenalk | Germany | 0 | Wilhelm Blasius. Der Riesenalk. Naumann, Naturgeschichte der Vögel Mitteleuropas. 1903;7. |
| Duke | Spain/France/  Newfoundland | 1 | W. A. Montevecchi, L. M. Tuck. Newfoundland birds: exploitation, study, conservation. Nuttall Ornithological Club. 1987.; Pierre Detchevery Dorre, 1677 |
| Esarokitsok/Isarukitsok/  Iserokitsok/Isorakitsoq | Greenland | 0 | Meldgaard M. The Great Auk, Pinguinus impennis (L.) in Greenland. Harwood Academic Publishers Inc. 1988;1(2):145–78.; Heinrich Christopher Glahns. Missionar i Gronland Heinrich Christopher Glahns Dagbogerfor Aarene 1763- 64, 1766-67 og 1767-68. H. Ostermann, editor. Vol. 4. Del Gronlandske Selskabs Skrifter; 1921. |
| Fiaert | Norway | 0 | Thomas Pennant. British Zoology [Internet]. 4th ed. Vol. 2. Warrington: William Eyres; 1761. Available from: https://doi.org/10.5962/bhl.title.62481 |
| F(l)ügloser Alk | Denmark | 0 | Henry Eeles Dresser. A history of the birds of Europe : including all the species inhabiting the western palaearctic region [Internet]. Vol. 8. London: Henry Eeles Dresser; 1881. Available from: https://doi.org/10.5962/bhl.title.53765 |
| Gaarfugl(ur) | Faroe Islands | 0 | Jørgen Landt. Forsøg til beskrivelse over Færøerne. Faroe Islands: Trykt paa Tikjøbs forlag, hos Z. Breum; 1800. 479 p. |
| Gairfowl | St. Kilda, Scotland | 1 | Martin M. A description of the Western Islands of Scotland. 2nd ed. London; 1703. |
| Garefowl(e) | Scotland/Republic of Ireland | 7 | Milne J. Relics of the great auk. London: Horace Cox; 1875.; Alexander Buchan. A Description of St. Kilda. 1773. |
| Garfågel | Sweden | 0 | Wilhelm Blasius. Der Riesenalk. Naumann, Naturgeschichte der Vögel Mitteleuropas. 1903;7. |
| Garefogel | Germany/Faroe Islands/Sweden | 0 | Grieve S. The great auk, or garefowl (alca impennis, Linn.): Its history, archaeology, and remains. Edinburgh, Scotland: Grange Publishing Works; 1885. |
| Garfahl | Sweden | 0 | Wilhelm Blasius. Der Riesenalk. Naumann, Naturgeschichte der Vögel Mitteleuropas. 1903;7. |
| Garfowl | Scandinavia/Scotland | 0 | Grieve S. The great auk, or garefowl (alca impennis, Linn.): Its history, archaeology, and remains. Edinburgh, Scotland: Grange Publishing Works; 1885. |
| Garf(u/o)gel | Faroe Islands | 1 | Debes L. Færoæ & Færoa reserata: That is a description of the islands & inhabitants of Foeroe: Being seventeen islands subject to the King of Denmark, lying under 62 deg. 10 min. of North latitude : wherein several secrets of nature are brought to light, and some antiquities hitherto kept in darkness discovered [Internet]. London: F.L.; 1676. Available from: http://name.umdl.umich.edu/A37317.0001.001 |
| Gårfuglur | Faroe Islands | 0 | Wilhelm Blasius. Der Riesenalk. Naumann, Naturgeschichte der Vögel Mitteleuropas. 1903;7. |
| Gayrfowl | St. Kilda, Scotland | 0 | Wilhelm Blasius. Der Riesenalk. Naumann, Naturgeschichte der Vögel Mitteleuropas. 1903;7. |
| Gearrbhul | United Kingdom | 0 | Wilhelm Blasius. Der Riesenalk. Naumann, Naturgeschichte der Vögel Mitteleuropas. 1903;7. |
| Geir | Iceland | 0 | Horrebow N. The Natural History of Iceland. London; 1758. |
| Geirfugl | Scandinavia | 0 | Snorri Sturluson. Prose Edda. Vol. 2. Copenhagen; 1848. 488 p. |
| Geirvogel | Germany | 0 | Wilhelm Blasius. Der Riesenalk. Naumann, Naturgeschichte der Vögel Mitteleuropas. 1903;7. |
| Geyerfogel | Canada | 0 | John Francis Campbell. A short American tramp on the fall of 1864. Edinburgh: Edmonston and Douglas; 1865. |
| Geyrfugl | Iceland | 0 | In: Scottish Society of Antiquaries. 1871. p. 83. |
| Goi(r)fug(e)l | Faroe Islands/Scotland | 0 | Joannis Raii. Synopsis Methodica Avium & Piscium; Opus Posthumum quod Vivus recenfuit & perfecit ipfe infigniffimus Author: in Quo Multas Species, in ipfius Ornithologiâ & Ichthyologia defideratas, adjecit: Methodumque fuam Piscium Naturæ magìs convenientem reddidit. London; 1713.; Carolus Clusius. Exoticorum Decem Libri. Leyden; 1605. 367 p. |
| Gorfou | France | 0 | Wilhelm Blasius. Der Riesenalk. Naumann, Naturgeschichte der Vögel Mitteleuropas. 1903;7. |
| Gorfuglir | Faroe Islands | 0 | The Gare-fowl and its historians. The natural history review. 1865;12:475. |
| Le Grand Pingo(u)in/  Grand Pingouin du Nord | France | 0 | Thomas Pennant. British Zoology [Internet]. 4th ed. Vol. 2. Warrington: William Eyres; 1761. Available from: https://doi.org/10.5962/bhl.title.62481; George Edwards. A Natural History of Birds. Vol. 3. London: College of Physicians in Warwick-Lane; 1750.; Georges Louis Leclerc Buffon. Histoire naturelle des oiseaux. Vol. 9. Paris: Imprimerie Royale; 1779.; Preyer W. Ueber Plautus impennis (Alca impennis L.). A. Emmerling; 1862. |
| Great Auk | Entire range | 6 | Thomas Pennant. British Zoology [Internet]. 4th ed. Vol. 2. Warrington: William Eyres; 1761. Available from: https://doi.org/10.5962/bhl.title.62481 |
| Grosser Alk | Germany | 0 | Wilhelm Blasius. Der Riesenalk. Naumann, Naturgeschichte der Vögel Mitteleuropas. 1903;7. |
| Grosser Papageitaucher | Germany | 0 | Wilhelm Blasius. Der Riesenalk. Naumann, Naturgeschichte der Vögel Mitteleuropas. 1903;7. |
| G(e)yrfugl/Gyrvgeyrfugl | Iceland | 0 | Thomas Pennant. British Zoology [Internet]. 4th ed. Vol. 2. Warrington: William Eyres; 1761. Available from: https://doi.org/10.5962/bhl.title.62481 |
| Havtirdill | Iceland | 0 | Wilhelm Blasius. Der Riesenalk. Naumann, Naturgeschichte der Vögel Mitteleuropas. 1903;7. |
| King and Queen of the Auks | Papa Westra, Orkney, Scotland | 1 | Nettleship DN, Evans PGH. The Atlantic Alcidae: the evolution, distribution, and biology of the auks inhabiting the Atlantic Ocean and adjacent water areas. Distribution and Status of the Atlantic Alcidae. 1985;54–154. |
| Kurzflügeliger Alk/  Kurzflügeliger | Germany | 0 | Wilhelm Blasius. Der Riesenalk. Naumann, Naturgeschichte der Vögel Mitteleuropas. 1903;7. |
| Large Auk | Newfoundland | 0 | Richard Henry Bonnycastle. Newfoundland in 1842: A Sequel to “The Canadas in 1841.” Vol. 2. London: Henry Colburn; 1842. |
| Lengurn | Canada | 1 | Grieve S. The great auk, or garefowl (alca impennis, Linn.): Its history, archaeology, and remains. Edinburgh, Scotland: Grange Publishing Works; 1885. |
| Matœoptera impennis | Sweden | 0 | Preyer W. Ueber Plautus impennis (Alca impennis L.). A. Emmerling; 1862. |
| Mergus Americanus | North America | 0 | Carolus Clusius. Exoticorum Decem Libri. Leyden; 1605. 367 p. |
| Moyack | Massachusetts/Labrador | 0 | Baron de Lahontan. New voyages to North America. La Haye; 1703. 61 p. |
| Noordscher Penguyn | Netherlands | 0 | Wilhelm Blasius. Der Riesenalk. Naumann, Naturgeschichte der Vögel Mitteleuropas. 1903;7. |
| Nordischer Fettgans/  Nordischer Pinguin | Germany | 0 | Wilhelm Blasius. Der Riesenalk. Naumann, Naturgeschichte der Vögel Mitteleuropas. 1903;7. |
| Nordske Penguin | Sweden | 0 | Wilhelm Blasius. Der Riesenalk. Naumann, Naturgeschichte der Vögel Mitteleuropas. 1903;7. |
| Northern Auk | United Kingdom | 0 | Preyer W. Ueber Plautus impennis (Alca impennis L.). A. Emmerling; 1862. |
| Northern Penguin | Unknown | 0 | Wilhelm Blasius. Der Riesenalk. Naumann, Naturgeschichte der Vögel Mitteleuropas. 1903;7. |
| Ogaen | Unknown | 0 | Wilhelm Blasius. Der Riesenalk. Naumann, Naturgeschichte der Vögel Mitteleuropas. 1903;7. |
| Peegvie | Norway | 0 | Wilhelm Blasius. Der Riesenalk. Naumann, Naturgeschichte der Vögel Mitteleuropas. 1903;7. |
| Penguin | Entire range | 24 | Archer G. Hakluytus Posthumus or, Purchas his Pilgrimes: Contayning a History of the World in Sea Voyages and Lande Travells by Englishmen and Others [Internet]. 4th ed. Vol. 18. Hakluyt Society; 1602 [cited 2023 Sep 2]. Available from: https://www.cambridge.org/core/product/identifier/9781316050798/type/book; Grieve S. The great auk, or garefowl (alca impennis, Linn.): Its history, archaeology, and remains. Edinburgh, Scotland: Grange Publishing Works; 1885.; Morey DC. The Voyage of Archangell. Gardiner, ME: Tilbury House Publishers; 2005.; Eckstrom F (Hardy). Testimony of Some Early Voyagers on the Great Auk. The Auk. 1888;5(4). |
| Penguyn | Netherlands | 0 | Preyer W. Ueber Plautus impennis (Alca impennis L.). A. Emmerling; 1862. |
| Pengwin | Cape Cod, Massachusetts | 3 | Archer G. Hakluytus Posthumus or, Purchas his Pilgrimes: Contayning a History of the World in Sea Voyages and Lande Travells by Englishmen and Others [Internet]. 4th ed. Vol. 18. Hakluyt Society; 1602 [cited 2023 Sep 2]. Available from: https://www.cambridge.org/core/product/identifier/9781316050798/type/book |
| Pengwyn | Cape Breton and Funk Island, Canada | 2 | Forbush EH. A history of the game birds, wild fowl and shore birds of Massachusetts and adjacent states, including those used for food which have disappeared since the settlement of the country, and those which are now hunted for food or sport, with observations on their former abundance and recent decrease in numbers; also the means for conserving those still in existence [Internet]. 2nd ed. Boston: Wright & Potter Printing Company, State Printers; 1916. Available from: https://doi.org/10.5962/bhl.title.15679 |
| Pennego(u)in | Newfoundland, Canada | 1 | Wilhelm Blasius. Der Riesenalk. Naumann, Naturgeschichte der Vögel Mitteleuropas. 1903;7. |
| Penngwyne | Newfoundland, Canada | 1 | Gaskell J. Who Killed the Great Auk? OUP Oxford; 2001. |
| Pingouin (brachiptère) | France | 0 | Forbush EH. A history of the game birds, wild fowl and shore birds of Massachusetts and adjacent states, including those used for food which have disappeared since the settlement of the country, and those which are now hunted for food or sport, with observations on their former abundance and recent decrease in numbers; also the means for conserving those still in existence [Internet]. 2nd ed. Boston: Wright & Potter Printing Company, State Printers; 1916. Available from: https://doi.org/10.5962/bhl.title.15679 |
| Pinguin-Alk | Germany | 0 | Wilhelm Blasius. Der Riesenalk. Naumann, Naturgeschichte der Vögel Mitteleuropas. 1903;7. |
| Pinguinus impennis | Entire range | 0 | Pierre Joseph Bonnaterre. Tableau encyclopédique et méthodique des trois règnes de la nature Ornithologie [Internet]. Paris; 1823. Available from: https://doi.org/10.5962/bhl.title.119325 |
| Pingwin | Faroe Islands | 0 | Grieve S. The great auk, or garefowl (alca impennis, Linn.): Its history, archaeology, and remains. Edinburgh, Scotland: Grange Publishing Works; 1885. |
| Pinwing | Newfoundland | 0 | Forbush EH. A history of the game birds, wild fowl and shore birds of Massachusetts and adjacent states, including those used for food which have disappeared since the settlement of the country, and those which are now hunted for food or sport, with observations on their former abundance and recent decrease in numbers; also the means for conserving those still in existence [Internet]. 2nd ed. Boston: Wright & Potter Printing Company, State Printers; 1916. Available from: https://doi.org/10.5962/bhl.title.15679 |
| Plautus impennis | Sweden | 0 | Preyer W. Ueber Plautus impennis (Alca impennis L.). A. Emmerling; 1862. |
| Reuzenalk | Netherlands | 0 | Wilhelm Blasius. Der Riesenalk. Naumann, Naturgeschichte der Vögel Mitteleuropas. 1903;7. |
| Schwimmalk | Germany | 0 | Wilhelm Blasius. Der Riesenalk. Naumann, Naturgeschichte der Vögel Mitteleuropas. 1903;7. |
| Sea-Woggin/Waggin | Newfoundland | 0 | Olson SL, Lund JN. Whalers and woggins: a new vocabulary for interpreting some early accounts of the great auk and penguins. Archives of Natural History. 2007;34(11):69–78. |
| Storömmer | Norway | 0 | Wilhelm Blasius. Der Riesenalk. Naumann, Naturgeschichte der Vögel Mitteleuropas. 1903;7. |
| Stummelalk | Germany | 0 | Wilhelm Blasius. Der Riesenalk. Naumann, Naturgeschichte der Vögel Mitteleuropas. 1903;7. |
| Tangueu/Tangueux | Canada | 1 | Wilhelm Blasius. Der Riesenalk. Naumann, Naturgeschichte der Vögel Mitteleuropas. 1903;7. |
| Vulture | Iceland | 5 | Horrebow N. The Natural History of Iceland. London; 1758. |
| Waggin/Wargan/  Wargin/Waugin | North America | 2 | Olson SL, Lund JN. Whalers and woggins: a new vocabulary for interpreting some early accounts of the great auk and penguins. Archives of Natural History. 2007;34(11):69–78. |
| W(h)obble | New England | 1 | Josselyn J. New England’s rarities discovered. London; 1672. |
| Wogæn/Wogen/Woggin/  Woging/Woglin | North America | 2 | Olson SL, Lund JN. Whalers and woggins: a new vocabulary for interpreting some early accounts of the great auk and penguins. Archives of Natural History. 2007;34(11):69–78. |
| Wuttoowaganash | Squibnocket, Massachusetts | 0 | Forbush EH. A history of the game birds, wild fowl and shore birds of Massachusetts and adjacent states, including those used for food which have disappeared since the settlement of the country, and those which are now hunted for food or sport, with observations on their former abundance and recent decrease in numbers; also the means for conserving those still in existence [Internet]. 2nd ed. Boston: Wright & Potter Printing Company, State Printers; 1916. Available from: https://doi.org/10.5962/bhl.title.15679 |
| Yawgin | North America | 0 | Olson SL, Lund JN. Whalers and woggins: a new vocabulary for interpreting some early accounts of the great auk and penguins. Archives of Natural History. 2007;34(11):69–78. |
